# Diagnostic and prognostic value of (cone-beam) computed tomography in dental sleep medicine for obstructive sleep apnea: a systematic review

**DOI:** 10.1186/s13005-026-00609-x

**Published:** 2026-03-12

**Authors:** Janine Sambale, Janine Hass, Ulrich Koehler, Susanne Waldmann, Heike Maria Korbmacher-Steiner

**Affiliations:** 1 Department of Orthodontics, Clinic of Dentistry, Marburg University, Georg-Voigt-Str. 3, Marburg, 35039 Germany; 2 Department of Pneumology, Interdisciplinary Center of Sleep Medicine, Marburg University, Baldingerstr, Marburg, 35043 Germany; 3 Central Medical Library, University Library, Marburg University, Conradistr. 3a, Marburg, 35032 Germany

**Keywords:** Obstructive sleep apnea, Cone-beam computed tomography, Polysomnography, Dental sleep medicine, Diagnostic

## Abstract

**Study Objectives:**

Determine whether cone-beam computed tomography (CBCT) or computed tomography (CT) measurements of the upper airway, craniofacial structures and soft tissues provide diagnostic or prognostic value for obstructive sleep apnea (OSA) in dental sleep medicine.

**Methods:**

Following PRISMA guidelines, a comprehensive search was conducted in PubMed, Embase, Cochrane Library, Web of Science and ClinicalTrials.gov. Eligible studies until September 2025 included polysomnography or polygraphy confirmed OSA, CBCT- or CT-based measurements, and either PSG/PG-confirmed non-OSA controls, defined OSA severity strata or treatment responder classifications. Data were extracted for diagnostic and therapeutic studies and risk of bias was assessed with the QUIPS tool.

**Results:**

Of 392 records, 11 met inclusion criteria (7 diagnostic, 4 therapeutic). Most were subject to high risk of bias and showed heterogeneity in imaging protocols, measurements and outcome definitions, with little adjustment for age, gender or body mass index. Individuals with OSA often had smaller retropalatal minimum cross-sectional area (CSAmin) whereas findings for retroglossal dimensions, total airway volume, skeletal morphology and soft-tissue measures were inconsistent and lacked clear correlation with disease severity. In therapeutic studies, upper-airway volume generally increased, but baseline CBCT- and CT-derived variables and treatment-related airway enlargement did not reliably distinguish responders from non-responders.

**Conclusions:**

Available evidence does not support CBCT- and CT-derived airway, skeletal or soft-tissue parameter as useful tools for diagnosing and grading of OSA or predicting treatment response. Given the high radiation exposure associated with both modalities, routine CBCT or CT solely for OSA screening or outcome prediction in dental sleep medicine is not justified and should be limited to established guidelines or surgical planning.

**Trial registration:**

PROSPERO ID CRD42024556837.

**Supplementary Information:**

The online version contains supplementary material available at 10.1186/s13005-026-00609-x.

## Introduction

Obstructive sleep apnea (OSA) is a common sleep-related breathing disorder that affects both adults and children. It is characterized by recurrent episodes of upper airway obstruction during sleep, which lead to intermittent hypoxemia, cortical arousals and sleep fragmentation. With an estimated global prevalence of almost one billion people, OSA is a significant public health concern due to its well-documented links with cardiovascular disease, stroke, diabetes, impaired cognitive function, and excessive daytime sleepiness [1–4].

OSA is diagnosed through either full-night polysomnography (PSG), the diagnostic gold standard, or through home sleep testing (polygraphy) [5]. Disease severity is quantified using the apnea-hypopnea index (AHI), which is defined as the number of apneas and hypopneas per hour of sleep. According to the international consensus criteria, OSA is classified as mild (5 ≤ AHI < 15), moderate (15 ≤ AHI ≤ 30) or severe (AHI > 30). Treatment is generally recommended for patients with moderate-to-severe disease or for those with symptomatic mild OSA [1, 6]. In addition to PSG or polygraphy (PG), validated screening instruments – such as the Epworth Sleepiness Scale (ESS) and the STOP-BANG questionnaire – are widely used to identify individuals at risk and guide referral for diagnostic evaluation [7, 8].

The pathophysiology of OSA involves a complex interplay of anatomical and non-anatomical traits affecting upper airway stability during sleep. Anatomical factors, such as obesity-related soft-tissue crowding, retrognathia, maxillary constriction, macroglossia and, in children, enlarged tonsils and adenoids, increase airway collapsibility [9]. Non-anatomical factors, such as impaired upper airway muscle responsiveness, high loop gain and a low arousal threshold, further influence disease severity [10]. As many of these characteristics are craniofacial in origin, dentists and orthodontists are well positioned to recognize risk factors for OSA. This highlights their increasing involvement in screening and interdisciplinary management [11].

Continuous positive airway pressure (CPAP) therapy remains the first-line treatment for OSA and is highly effective in normalizing AHI; however, long-term adherence is often limited due to discomfort and inconvenience [12]. Within an interdisciplinary orthodontic-maxillofacial framework, surgical interventions – most notably maxillomandibular advancement (MMA) and surgically assisted expansion for transverse maxillary deficiency – provide causal treatment options by enlarging the upper airway. These procedures are particularly indicated for young patients with pronounced craniofacial anomalies (e.g., Pierre Robin sequence, Crouzon syndrome, Apert syndrome) or skeletal deficiencies such as micrognathia or mandibular retrognathia but may also be effective in normognathic individuals [13, 14]. Mandibular advancement devices (MADs) are considered a validated second-line therapy, especially for patients with mild-to-moderate OSA or intolerance to CPAP [15–17]. MADs protrude the mandible during sleep, thereby enlarging upper-airway dimensions and reducing pharyngeal collapsibility [18]. Nevertheless, treatment response varies widely, with approximately 40–60% of patients achieving clinically meaningful improvement, depending on the definition applied [19]. The growing contribution of dental specialists to the management of OSA further highlights the relevance of craniofacial characteristics in predicting MAD treatment outcome [20].

Cone-beam computed tomography (CBCT) has become widely used in dentistry for three-dimensional evaluation of craniofacial structures and is increasingly applied to upper-airway assessment. CBCT and computed tomography (CT) enable volumetric analysis of the upper airway, including total volume, minimum cross-sectional area (CSAmin), and site-specific constrictions. Several studies have reported smaller airway volumes or more constricted retropalatal or retroglossal regions in patients with OSA compared with healthy controls, suggesting a potential anatomical predisposition [21].

However, the diagnostic and prognostic value of CBCT and CT in OSA remains uncertain. In its 2011 position statement, the American Academy of Oral and Maxillofacial Radiology (AAOMR) already emphasized that CBCT-derived airway measurements lacked validated diagnostic accuracy and provided no demonstrated relevance for therapeutic decision-making. The AAOMR highlighted a substantial research gap and called for rigorous investigation into whether CBCT offers any measurable diagnostic or therapeutic benefit in orthodontic and airway-related applications, particularly with respect to clinical decision-making and patient outcomes [22].

A number of systematic reviews have previously investigated whether craniofacial characteristics may serve as indicators of OSA. However, many of these reviews included presumed non-OSA control groups in whom an AHI < 5 had not been confirmed by PSG- or PG-confirmed non-OSA, thereby considerably limiting the validity of their conclusions [21, 23–25]. Taha et al. [25] included only studies with individuals with OSA and assessed the correlation of severity degrees of OSA and the AHI. The authors concluded that the CSA and upper airway measured by CBCT were weak predictors of OSA.

The diagnostic and prognostic value of CBCT or CT in OSA remains uncertain; however, narrowed airway spaces on CBCT are frequently interpreted in dental practice as indicative of OSA. CBCT- or CT-derived measurements are often used to predict potential treatment response. Due to the fact that CBCT – and particularly CT – exposes patients to considerably higher radiation doses in comparison with conventional two-dimensional imaging, its utilization is required to adhere to the ALARA principle (“as low as reasonably achievable”), and each exposure must be clinically justified.

Given the increasing application of CBCT in dental sleep medicine for OSA screening and treatment planning - particularly in the context of oral appliance therapy, orthodontic interventions, and interdisciplinary surgical treatment concepts - a systematic evaluation of its diagnostic and intervention-related utility is necessary.

The present systematic review therefore aimed to determine whether CBCT- or CT-based airway, craniofacial, or soft-tissue measurements can (1) distinguish between individuals with and without OSA, (2) diagnose disease severity, or (3) predict treatment response.

## Materials and methods

This systematic review was carried out in accordance with the Preferred Reporting Items for Systematic Reviews and Meta-Analyses (PRISMA) guidelines and was prospectively registered in the International Prospective Register of Systematic Reviews (PROSPERO; identifier: CRD42024556837, registration date: June 21, 2024) [26].

### Search strategy

A comprehensive electronic search was conducted in four databases and study registries were searched:

- PubMed (MEDLINE including MEDLINE In-Process) (1948 to September 01, 2025)
- Embase.com (Elsevier) (1974 to September 01, 2025)
- Cochrane Central Register of Cochrane Reviews and Controlled Trials (CENTRAL) (1992 to September 01, 2025)
- Web of Science Core Collection (WOS.SCI: 1900 to 2025; WOS.AHCI: 1975 to 2025; WOS.ESCI: 2020 to 2025; WOS.SSCI: 1956 to 2025) (to September 01, 2025)
- ClinicalTrials.gov (to September 01, 2025)

The strategy combined vocabulary (e.g., MeSH, Emtree) and free-text terms related to three main domains: OSA, CBCT and dentistry. Search terms were refined through preliminary searches. The final search terms were combined with AND across domains and OR within domains with syntax adapted to individual databases (see Appendix I).

### Study selection

Eligibility criteria were prespecified by the authors and defined according to whether studies assessed the diagnostic or therapeutic value of CBCT in dentistry among patients suspected to OSA.

Inclusion criteria for diagnostic studies were as follows: subjects with OSA confirmed by sleep recordings, use of CBCT or CT to evaluate the posterior airway space or other craniofacial/orofacial parameters, and provision of comparative data either between patients with and without OSA or between groups stratified by OSA severity.

Inclusion criteria for therapeutic studies were: performance of both CBCT or CT and sleep recordings before and after an intervention (e.g., oral appliance therapy, upper-airway surgery, orthodontic treatment); assessment of the posterior airway space or other craniofacial/orofacial parameters, and the presence of comparator groups, defined as patients with versus without OSA, patients stratified by OSA severity, or responders versus non-responders following intervention. Exclusion criteria comprised studies with fewer than 10 participants per group (OSA and comparator groups), magnetic resonance imaging, studies involving patients with craniofacial or genetic syndromes, case reports, narrative reviews, systematic reviews, letters, editorials, surveys, and questionnaire-based studies. Experimental, cadaveric, or animal studies, as well as studies relying exclusively on machine-learning approaches without clinical data, were also excluded. All articles were limited to English and German languages. All retrieved records were exported into Zotero reference management software.

Deduplication of citations was performed within Zotero. The software’s *tag* function was used to document screening decisions at each stage (title/abstract and full-text). Tags were independently applied by two authors (JS, JH) to indicate inclusion, exclusion, or “unclear,” ensuring transparent tracking of decisions. Both authors (JS, JH) independently screened the titles and abstracts of all records identified through the search strategy. Full texts of potentially eligible studies were retrieved and assessed independently against the predefined inclusion and exclusion criteria. At each step a third author (HMKS) controlled the collection and confirmed the final articles included. Any disagreements at either stage were resolved through discussion between the authors (JS, JH, HMKS). All steps of the selection process were documented in a PRISMA flow diagram.

### Data collection

Two authors (JS, JH) conducted independent data extraction from the full texts, which was then reviewed by the entire author team. Data derived from questionnaires, clinical examinations, digital models and rhinomanometry were excluded from the analysis. Substantial heterogeneity was identified during the extraction process in both sleep-related parameters and CBCT- and CT-derived measurements. In particular, CBCT and CT variables were reported using inconsistent terminology, and the types of measurements recorded varied considerably across studies. Due to the lack of standardized definitions for CBCT and CT variables, each publication was carefully reviewed to ensure the comparability of reported measurements. All extracted sleep parameters and CBCT and CT measurements are summarized in supplementary material (Appendix II–V), where abbreviations, alternative designations and corresponding definitions are provided for each variable.

The extracted data were categorized as either diagnostic or therapeutic studies. Table 1 summarizes the studies investigating the diagnostic value of CBCT or CT in the assessment of OSA. The following information was recorded for each study:

**Table 1:** Overview of the main characteristics and results of the included studies: diagnostic of OSA by CBCT and CT

Study and country	Sample size and research design	Grouping and group sizes	Gender	Mean age (in years)	Mean BMI (in kg/m^2^)	Mean AHI/RDI	Diagnostic intervention	Results relevant to this systematic review
Chen et al. (2002) [28] China	*n* = 117 prospective	Snorers: RDI < 5 (*n* = 19) OSA: RDI > 5 (*n* = 98)	Snorers: female: *n* = 3 male: *n* = 16 OSA: female: *n* = 9 male: *n* = 89	Snorers: 45.21 ± 12.14 OSA: 44.78 ± 11.48	Snorers: 26.97 ± 3.34 OSA: 27.33 ± 3.99	Snorers: RDI: 2.33 ± 1.48 OSA: RDI: 41.48 ± 26.45	One-night PSG: sleep parameters: RDI, minimal O_2_ saturation, snore index, percentage of slow wave sleep in sleep period total, REM Supine CT during quit expiratory phase: retropalatal region: anterioposterior and lateral diameters of CSAmin, CSAmin; retroglossal region: CSAmin; total volume of the retropalatal and retroglossal region	The retroglossal airway was found to be similar in snorers and subjects with OSA, while the retro-palatal area showed a significant difference between the two groups. Retropalatal airway narrowing was influenced by body weight. Subjects with OSA presented with a significantly higher body weight.
Ciavarella et al. (2025) [34] Italy	*n* = 40 retrospective	OSA severity: Moderate OSA: *n* = 21 Severe OSA: *n* = 19	Not reported.	Total sample size: 53.5 years In groups not reported.	Inclusion criteria: < 33.9 In groups not reported.	Moderate OSA: AHI: 22.78 Severe OSA: AHI: 45.37	One-night PSG: AHI, ODI Upright CBCT: Total airway volume, CSAmin, anterioposterior and lateral diameters of CSAmin	Subjects with increasing OSA severity exhibited progressively reduced total airway volume and CSAmin. The narrowing was most pronounced in the retropalatal region. BMI and age were not reported between groups. There was no information about gender.
Enciso et al. (2010) [29] USA	*n* = 80 prospective	OSA severity: AHI < 10 (defined as snorers): *n* = 34 AHI ≥ 10 (defined as patients with OSA): *n* = 46	Total sample size: female: *n* = 17 male: *n* = 63 Snorers: female: *n* = 13 male: *n* = 21 OSA: female: *n* = 4 male: *n* = 42	Snorers: 50.8 ± 13.46 OSA: 57.5 ± 10.25	Snorers: 25,13 ± 3,33 OSA: 27.7 ± 3.83	Snorers: AHI: 4.3 ± 2.85 OSA: AHI: 27.6 ± 17.13	Two-night PG: AHI, AI Supine CBCT: CSAmin, anterior-posterior and lateral diameters of CSAmin, airway length, vertical and horizontal soft palate dimensions, total airway volume, uniformity of volume	No statistically significant differences in airway volume or CSAmin were detected between subjects with OSA and controls. Variability within the OSA group limited the ability to distinguish severity-related patterns.
Enciso et al. (2012) [30] USA	86 prospective	RDI < 15 (defined as mild OSA) RDI ≥ 15 (defined as moderate to severe OSA patients)	Total sample size: female: *n* = 20 male: *n* = 66 Mild OSA: female: *n* = 12, male: *n* = 21 Moderate to severe OSA: female: *n* = 8 male: *n* = 45	Mild OSA: 47.6 ± 12.74 Moderate to severe OSA: 58.4 ± 10.35	Mild OSA: 25.0 ± 3.65 Moderate to severe OSA: 27.6 ± 3.74	Mild OSA: AHI: 1.2 ± 1.48, RDI: 8.0 ± 3.61 Moderate to severe OSA: AHI: 14.2 ± 15.5, RDI: 33.9 ± 16.3	One-night PG: AHI, RDI Supine CBCT: evaluation of the location, nature, and occurrence of incidental findings in the airway and maxillofacial structures (ethmoidal/maxillary sinus, nasal findings, tonsil findings, airway narrowing, soft palate/tongue findings, focal calcifications, TMD)	Compared with patients with mild OSA, those with moderate to severe OSA more frequently presented with concha bullosa, turbinate hyper-trophy, tonsillar enlargement, an elongated or posteriorly positioned soft palate, a narrower airway, macroglossia, and focal calcifications. In contrast, ethmoidal or maxillary sinus abnormalities and temporomandibular joint findings were more prevalent in the mild OSA group than in moderate to severe cases. However, the examination was qualitative and most of these differences did not reach statistical significance.
Firincioglulari et al. (2020) [31] Cyprus	198 retrospective	Controls: (no OSA symp-toms): *n* = 99 AHI < 5 (minimal OSA): *n* = 22 AHI 5–15 (mild OSA): *n* = 21 AHI 15–30 (moderate OSA): *n* = 19 AHI > 30 (severe OSA): *n* = 35	Not reported.	Controls: 46.6 OSA: 53.8	Controls: not reported. OSA: 33.9	OSA: AHI: 27.3	One-night PSG: AHI Supine CBCT: genial tubercles and lingual foramina were examined in sagittal, coronal and axial views.	In individuals diagnosed with OSA, the anterior mandibular cortex was found to be thicker, while the genial tubercle appeared narrower. Further-more, the vertical distance from the lingual foramen to the inferior border of the mandible was found to be significantly greater in the OSA group. However, no significant differences were observed between the groups in terms of the number or frequency of lingual foramina, nor in their anatomical location. The vertical distance of the lingual foramen to the lower border of the mandible, BMI and age showed a statistically significant association with the AHI.
Lam et al. (2003) [32] China	92 prospective	Controls (*n* = 36): AHI < 5 AHI 5–30 (mild/ moderate OSA): *n* = 34 AHI > 30 (severe OSA): *n* = 22	Total sample size: female: *n* = 23 (25%) male: *n* = 69 (75%)	Controls: 41.5 ± 6.4 Mild/ moderate: 45.8 ± 8.2 Severe: 47.2 ± 9.1	Controls: 24.2 ± 3.2 Mild/ moderate: 26.8 ± 3.5 Severe: 27.4 ± 3.4	Controls: AHI: 1.5 ± 1.3 Mild/moderate: AHI: 15.7 ± 6.5 Severe: AHI: 38.5 ± 6.5	One-night PSG: AHI Supine CT: cephalometric variables (MPH, SNA, SNB, ANB, PMU, Max. SP), CSA of the airway at the level of the velopharynx (tip of uvula) and hypopharynx (floor of vallecula)	Compared with controls, individuals with OSA displayed a smaller velopharyngeal airway, a lower velopharynx-to-hypopharynx ratio, a more caudally positioned hyoid bone, an elongated and thickened soft palate, and a mandible more posteriorly positioned relative to the maxilla. After adjustment for BMI and age, those with severe OSA continued to show greater mandibular retroposition and increased soft palate length.
Schwab et al. (1993) [47] USA	42 prospective	Controls: RDI < 2: *n* = 15 RDI < 15 (snorers/mild OSA): *n* = 14 RDI > 15 (OSA): *n* = 13	Controls: female: *n* = 5 male: *n* = 10 Snorers/mild OSA: female: *n* = 2 male: *n* = 12 OSA: female: *n* = 0 male: *n* = 13	Controls: 32.0 Snorers/ mild OSA: 34.5 OSA: 38.0	Controls: 24.1 Snorers/ mild OSA: 28.6 OSA: 34.1	Controls: RDI: 0.0 Snorers/ mild OSA: RDI: 6.5 OSA: RDI: 26.0	One-night PSG: AHI, oxygen saturation Supine CT: upper airway: CSAmin of the nasopharynx and of two levels in the retropalatal (high and low), and of the retroglossal region (anatomical landmarks: hard and soft palate, tongue base, posterior pharyngeal wall). A facial mask was used as a diagnostic interface to correlate breathing physiology with imaging. The CT scanner’s slice acquisition signal was synchronized with the respiratory data.	Individuals with OSA demonstrated markedly reduced CSA in the retropalatal and retroglossal regions, as well as more pronounced dynamic airway narrowing throughout the respiratory cycle compared with controls. The greatest reduction in end-expiratory airway caliber was observed in the OSA cohort. However, individuals with OSA exhibited a significantly higher BMI and neck circumferences.

- Author, year and country of publication
- Total sample size and study design
- Gender
- Grouping strategy and group sizes
- Mean age
- Diagnostic intervention, including measurement methods
- Baseline AHI or RDI as outcome intervention
- Main results relevant to this systematic review

Therapeutic studies are summarized in Table 2. These studies evaluated the diagnostic and predictive value of CBCT or CT between individuals with and without OSA, as well as for identifying therapeutic responders and non-responders. In addition to the above variables, where available, the following data were extracted: age; BMI; AHI or RDI before treatment (t0); after treatment (t1); the diagnostic intervention at each time point and at follow up; and details of the therapeutic intervention.

**Table 2:** Overview of the main characteristics and results of the included studies: treatment of OSA and the role of CBCT and CT to predict treatment outcome

Study and country	Sample size and research design	Grouping and group sizes	Gender	Mean Age pre-(t0) and posttreatment (t1) (in years)	Mean BMI pre- (t0) and posttreatment (t1) (in kg/m^2^)	Mean AHI/RDI pre- (t0) and posttreatment (t1)	Diagnostic intervention pre- (t0) and posttreatment (t1) and follow-up time	Therapeutic intervention	Results relevant to this systematic review
Bariani et al. (2022)[43] Brazil	*n* = 24 prospective	Primary snoring: *n* = 13 OSA (AHI > 1 and SpO2 < 92%): *n* = 11	female: *n* = 8 male: *n* = 16	t0: 10.0 ± 1.8 t1: 11.3 ± 1.9	z-score for age: t0: 0.87 ± 2.16 t1: 0.37 ± 1.76	Snorers: t0: AHI: 0.83 ± 1.08; RDI: 0.94 ± 1.13 t1: AHI: 1.87 ± 3.65, RDI: 1.91 ± 3.64 OSA: t0: AHI: 2.26 ± 1.19; RDI: 2.37 ± 1.29 t1: AHI: 2.33 ± 1.41; RDI: 2.07 ± 1.30	One-night PSG: AHI, OAHI, CAHI, RDI, basal SpO2, mean SpO_2_, minimal SpO_2_, DI, mean saturation (awake, REM, Non-REM), total desaturation, sleep desaturation index Supine CT: upper airway: oropharynx volume	Hyrax palatal expander: activation protocol two turns per day (0.5 mm) till the palatal cusp of the upper molars was touching the buccal cusp of the lower molars. The device remained in place for six months.	A statistically significant increase in oropharyngeal airway volume was observed in both the primary snoring and OSA groups following rapid maxillary expansion. However, no significant correlation was found between the volumetric changes and alterations in oxygen saturation or AHI. This indicates that increase of airway volume does not directly translate into measurable improve-ments in polysomno-graphic parameters.
Chen et al. (2019)[42] Australia	*n* = 64 prospective	Responders (post-treatment AHI < 10 and AHI reduction of > 50%): *n* = 36 Non-responders (post-treatment AHI > 10 or AHI reduction < 50%): *n* = 28	Responders: female: *n* = 15 male: *n* = 21 Non-responders: female: *n* = 8 male: *n* = 20	Responders: 58.0 Non-responders: 59.0	Responders: 27.37 ± 3.62 Non-responders: 30.4 ± 5.64	Responders: t0: AHI: 22.2 t1: AHI: 4.05 Non-responders: t0: AHI: 26.9 t1: AHI: 13.5	One-night PSG: AHI Supine CBCT: Upper airway: CSAmin, volume of upper airway, average CSA, CSAmin/CSA, anterior-posterior and lateral dimension of CSAmin, upper airway length, surrounding structures: mandibular external length, maxillary length, soft palate length, area of the tongue, maxillomandibular enclosure size, anatomical balance (ratio) Follow-up time with MAD in situ: after 6 weeks	Mandibular advancement device (MAD: SomnoDent, SomnoMed^®^ Ltd, Sydney, Australia): The splints were constructed with 75% of maximum mandi-bular protrusion. Titration protocol: of 0.2 mm/day until the maximum comfortable level of the mandibular advancement was reached.	No significant difference in upper airway volume was identified between responders and non-responders following mandibular advancement therapy. CBCT-based volumetric parameters did not reliably predict treatment response, suggesting that airway morphology alone may not determine therapeutic success.
Pahkala et al. (2019)[46] Finland	*n* = 58 prospective	Complete responders: post-treatment AHI ≤ 5: *n* = 23 Partial-/Non-responders: partial response: AHI > 5 with AHI reduction > 50%, non-response: AHI > 5 with AHI reduction < 50%: *n* = 22	Complete responders: female: *n* = 7 male: *n* = 16 Partial-/Non-responders: female: *n* = 6 male: *n* = 16	Complete responders: 46.7 ± 13.3 Partial-/Non-responders: 54.8 ± 8.6	Complete responders: 28.0 ± 3.3 kg/m^2^ Partial-/Non-responders: 27.1 ± 2.8 kg/m^2^	Complete responders: t0: AHI: 18.4 ± 9.1 t1: AHI: 2.6 ± 1.7 Partial-/Non-responders: t0: AHI: 21.4 ± 8.2 t1: AHI: 10.1 ± 4.7	One-night PG: AHI, supine AHI, SaO_2_, percentage of SaO_2_ below 90%, proportion of sleep time spent snoring. Upright CBCT: Upper airway: volume and CSAmin of the nasopharynx, oropharynx and hypopharynx, total pharyngeal airway volume Follow-up time with MAD in situ: after 6 months	Mandibular advancement device (MAD: two-piece adjustable splint, SomnoDent Flex, SomnoMed Ltd., Sydney, Australia): The splints were constructed with 60% of maximum mandibular protrusion.	Complete responders exhibited greater changes in upper airway dimensions compared with partial or non-responders. Baseline upper airway characteristics between complete and partial/non-responders were not reported.
Thai et al. (2025)[35] Taiwan	*n* = 50 prospective	Responders (post-operative AHI < 10 and AHI reduction of > 50%): *n* = 30 Non-responders: *n* = 20	Responders: female: *n* = 11 male: *n* = 19 Non-responders: female: *n* = 3 male: *n* = 17	Responders: 32.9 ± 9.4 Non-responders: 31.8 ± 9.0	Responders: 22.0 ± 3.1 Non-responders: 23.0 ± 3.4	Responders: t0: AHI: 27.5 ± 20.1 t1: AHI: 3.5 ± 2.6 Non-responders: t0: AHI: 39.6 ± 20.9 t1: AHI: 17.9 ± 14.1	One-night PSG: AHI, CAI, minimum oxygen saturation Upright CBCT: skeletal: position/ inclination of the maxilla and mandible, ANB, mandibular and maxillary length/width/intergonial width/intercanine width, maxilla-mandibular volume upper airway: length, volume, anteroposterior and lateral diameter of CSAmin, CSAmin, surrounding structure: tongue length/height, soft palate length, maximum soft palate thickness, hyoid, anterior neck space volume Follow-up time: 2.0 ± 1.5 years	Modified maxillomandibular advancement (MMA): anterior segmental osteotomies combined with standard Le Fort I and bilateral sagittal split osteotomies, with or without mandibular counterclockwise rotation	Responders to MMA surgery had signi-ficantly lower pre-operative AHI values, a shorter maxillary length and a more superior-anterior hyoid position than non-responders. They also had shorter tongues and soft palates, as well as a wider oropharyngeal airway space. Preoperative maxillary and mandibular width, tongue length, airway length and extent of mandibular advancement were significant predictors of postoperative success.

### Risk of bias

The risk of bias (RoB) in the included studies was independently reassessed by two authors (JS, JH) and subsequently confirmed in consensus with the author team, using the Quality in Prognosis Studies (QUIPS) tool for assessing bias in prognostic factor research [27]. The QUIPS tool evaluates six domains: study participation, study attrition, prognostic factor measurement, outcome measurement, study confounding, and statistical analysis and reporting. Discrepancies between the two authors were resolved through discussion in the author team. The domain with the highest RoB determined the overall rating.

## Results

**Fig. 1:**
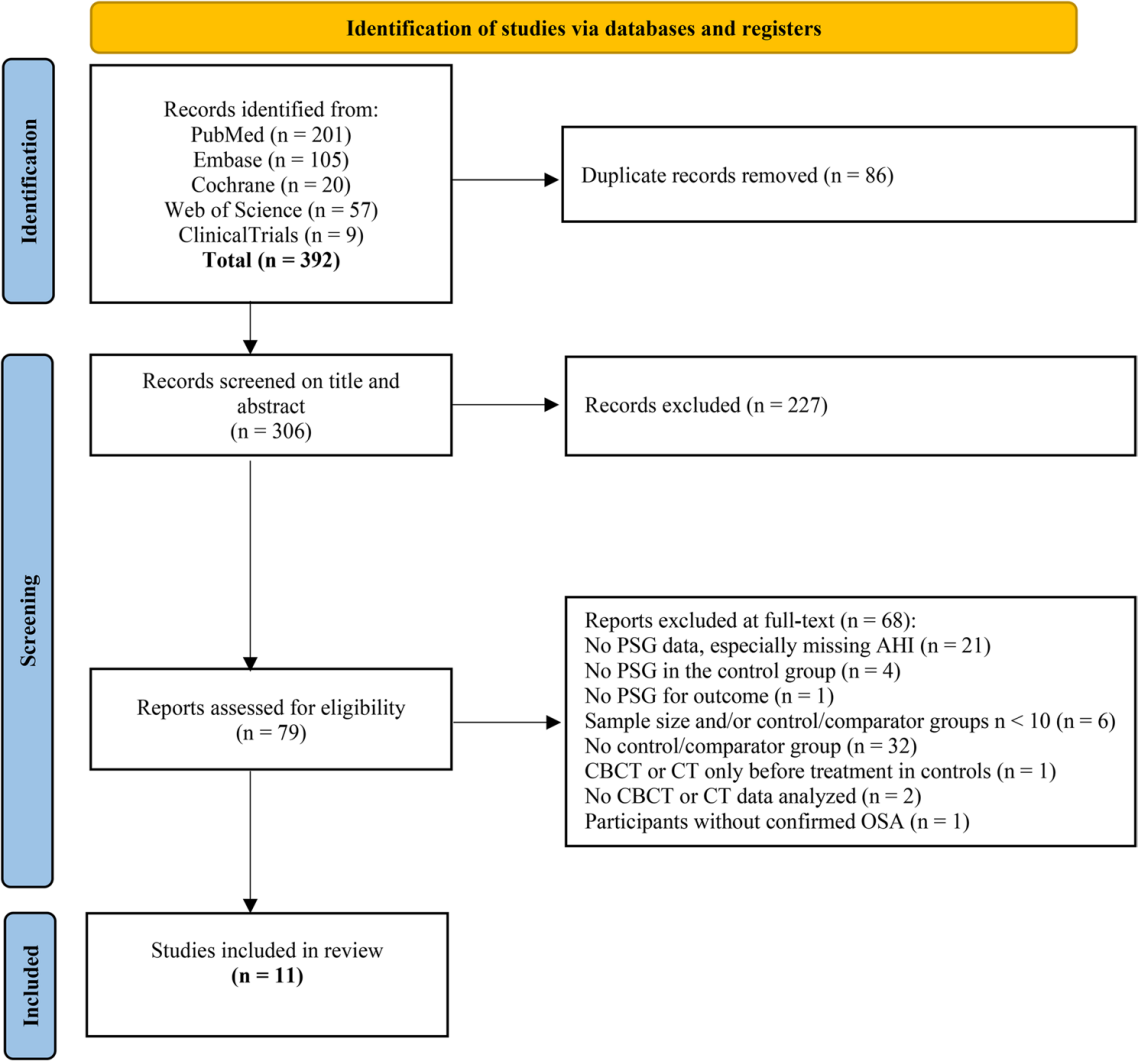
PRISMA flow chart of the article selection process for the systematic review of OSA, CBCT or CT and dentistry

A total of 392 records were identified in the initial search. After duplicates were removed, 306 studies underwent title and abstract screening. Of these, 79 were assessed in full text, and 11 met the inclusion criteria. The remaining 68 studies were excluded (see Fig. 1).

Seven [28–34] of the 11 included studies used CBCT or CT parameters for OSA screening (see Table 1), while four [35–38] investigated therapeutic interventions (see Table 2).

### Study characteristics

The 11 included studies were conducted across nine countries: China [28, 32], Italy [34], the USA [33, 39, 40], Cyprus [41], Brazil [36], Australia [37], Finland [38] and Taiwan [35].

Across all studies, the mean age of adults with OSA was 51.6 years (range 32–58 years), compared to 45.5 years in the control or primary snoring groups (mean difference + 6.1 years). One pediatric rapid maxillary expansion study reported a mean age of 10 years and was not included in the analysis of adult data [36].

The mean BMI of adult OSA groups was 27.6 kg/m² (range 24.5–34.1 kg/m²), compared to 24.5 kg/m² in the control or primary snoring groups (mean difference + 3.1 kg/m²). Severe OSA subgroups, where reported, were typically 2–4 kg/m² heavier than mild or moderate OSA groups. In therapeutic cohorts responders showed a slightly lower BMI than non-responders (a difference of ≤ 2 kg/m²) [35, 37, 38].

Male sex predominated across all adult cohorts. On average, 72% of individuals in OSA groups were male (range 60–100%), compared with 58% in control groups. No consistent difference in gender distribution was observed between therapeutic responders and non-responders.

Group sizes were approximately balanced in nine [32–36, 38, 39, 41, 42] and clearly unbalanced in two studies [28, 30].

### Risk of bias assessments

**Fig. 2:**
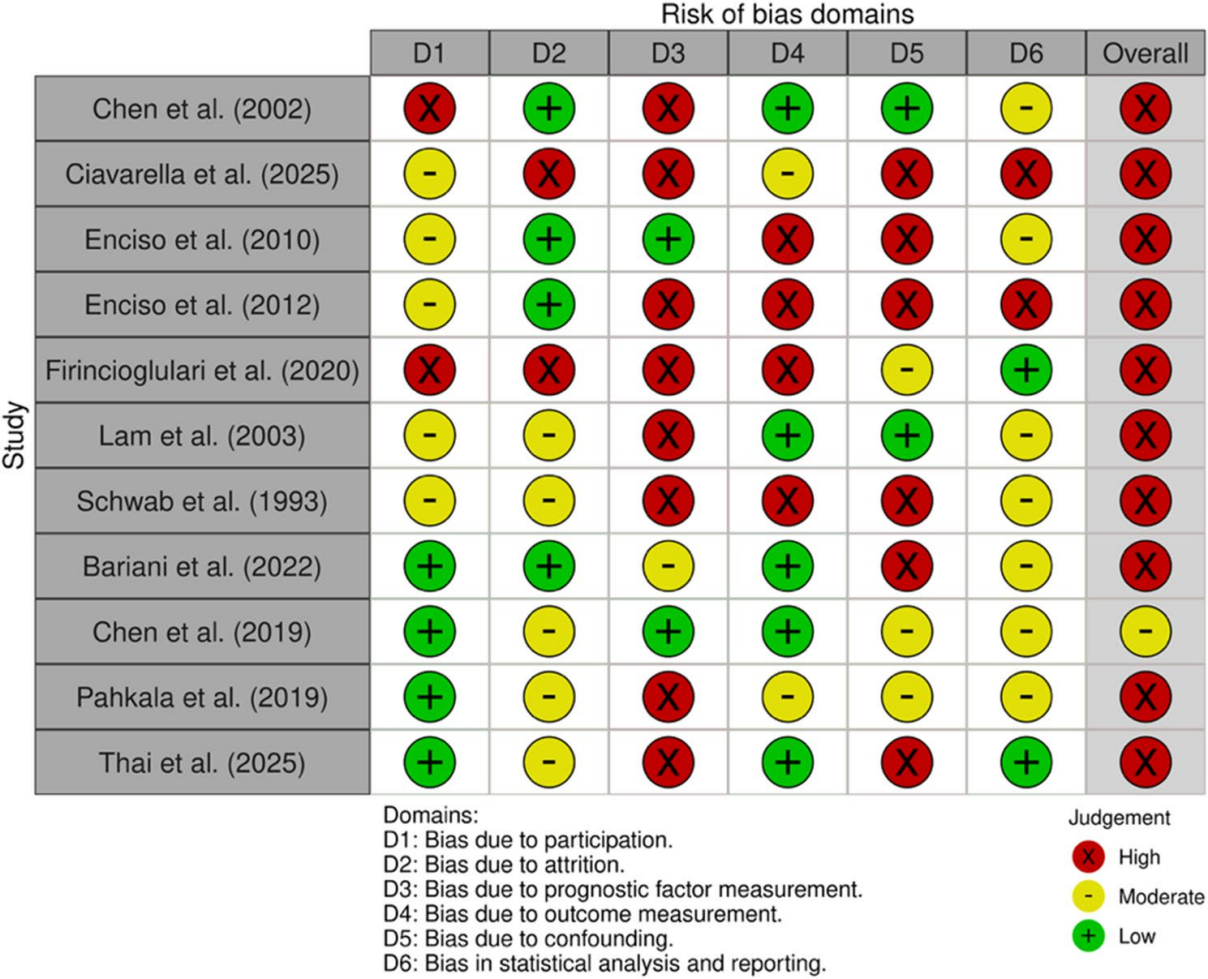
Risk-of-bias (RoB) domains and overall RoB

10 of the 11 studies were rated as being at high and one as being at moderate RoB overall (Fig. 2). In the study participation domain, 5 studies were judged to be at moderate RoB due to insufficient reporting of inclusion and exclusion criteria and/or missing details regarding the study setting and recruitment period. 2 studies were rated as being at high risk because no information was provided on the inclusion and exclusion criteria, the study location or the recruitment timeframe. For study attrition, 5 studies were rated as having a moderate RoB when no information was available on whether participants had been excluded during the study period. A high RoB was assigned if a complete, appropriate CONSORT-style flow diagram for retrospective studies was absent. In the prognostic factor measurement domain, 8 studies were rated as high risk due to the absence of blinded measurement procedures. 2 studies were rated as low risk because both intra- and inter-rater reliability were reported, and measurements were conducted under blinded conditions. 5 studies were judged as low risk for outcome measurement when polysomnography rather than polygraphy was used, and when signal acquisition and scoring followed the criteria established by the American Academy of Sleep Medicine. 4 studies were rated as high risk because the classification of OSA did not conform to guideline recommendations. 6 studies were assigned a high RoB regarding study confounding because key determinants of OSA - BMI, age and sex - were not accounted for in the analyses. Finally, 5 studies were rated as high risk in the statistical analysis and reporting domain when only simple t-tests were conducted, without applying regression models or estimating adjusted odds ratios that controlled for BMI, age and gender.

### Sleep characteristics

Sleep parameters used across the included studies are presented as supplementary material (Appendix II).

Sleep-disordered breathing was assessed using full PSG in eight studies [28, 32–35, 41, 43, 44] and portable polygraphy (PG) in three [38–40]. Only one study used two-night sleep monitoring; all the others used single-night recordings [39]. The AHI was the primary diagnostic metric in eight studies [34, 36–39, 41, 45]. Three studies defined OSA using the RDI [28, 33, 40].

However, group definitions were heterogeneous. Thresholds for individuals without OSA ranged from RDI < 2 [33] to AHI < 10 [39] while others used AHI or RDI < 5 [28, 32, 41]. One study [31] distinguished asymptomatic controls from individuals with mild OSA (AHI < 5), whereas others grouped habitual snorers with individuals presenting mild OSA [29, 33]. Responder classification in therapeutic cohorts was likewise inconsistent. Chen et al. [37] and Thai et al. [35] defined response as AHI < 10 combined with ≥ 50% reduction from baseline, whereas Pahkala et al. [46] applied a more stringent cut-off of AHI ≤ 5 for complete responders and classified partial responders separately.

Across the diagnostic cohorts (adults only), the mean baseline AHI was 33.5 in individuals with OSA and 7.0 in control or primary snoring groups (Δ = 26.5). The mean RDI was 27.4 in individuals with OSA versus 1.2 in comparators (Δ = 26.2). In therapeutic cohorts, baseline AHI averaged 22.7 in subsequent responders and 29.3 in non-responders. Post-treatment values declined to 3.4 in responders but remained elevated at 13.8 in non-responders.

### CBCT and CT measurements

Four studies [28, 33, 43, 45] performed CT scans, while seven studies [29–31, 34, 35, 44, 46] used CBCT. Eight studies [28–31, 33, 43–45] applied supine positioning, whereas three [34, 35, 46] conducted their measurements in the upright position.

The included studies comprised measurements of the upper airway (Appendix III), craniofacial structures (Appendix IV), and soft tissues (Appendix V). Nearly all studies (10 [28–30, 33–35, 43–46] of 11) assessed the upper airway; six studies [30, 31, 35, 43–45] (additionally) examined craniofacial structures, and four studies [29, 30, 35, 44] included soft-tissue measurements.

Across studies, measurement approaches varied substantially. Some investigations [28, 32] relied on manual 2D point marking and tracing of airway contours on CT or CBCT slices, whereas others [29, 30, 33–36] used software-based semiautomatic or automatic segmentation tools. One study [30] reported qualitative rather than quantitative assessments.

### Upper airway dimension

**Fig. 3:**
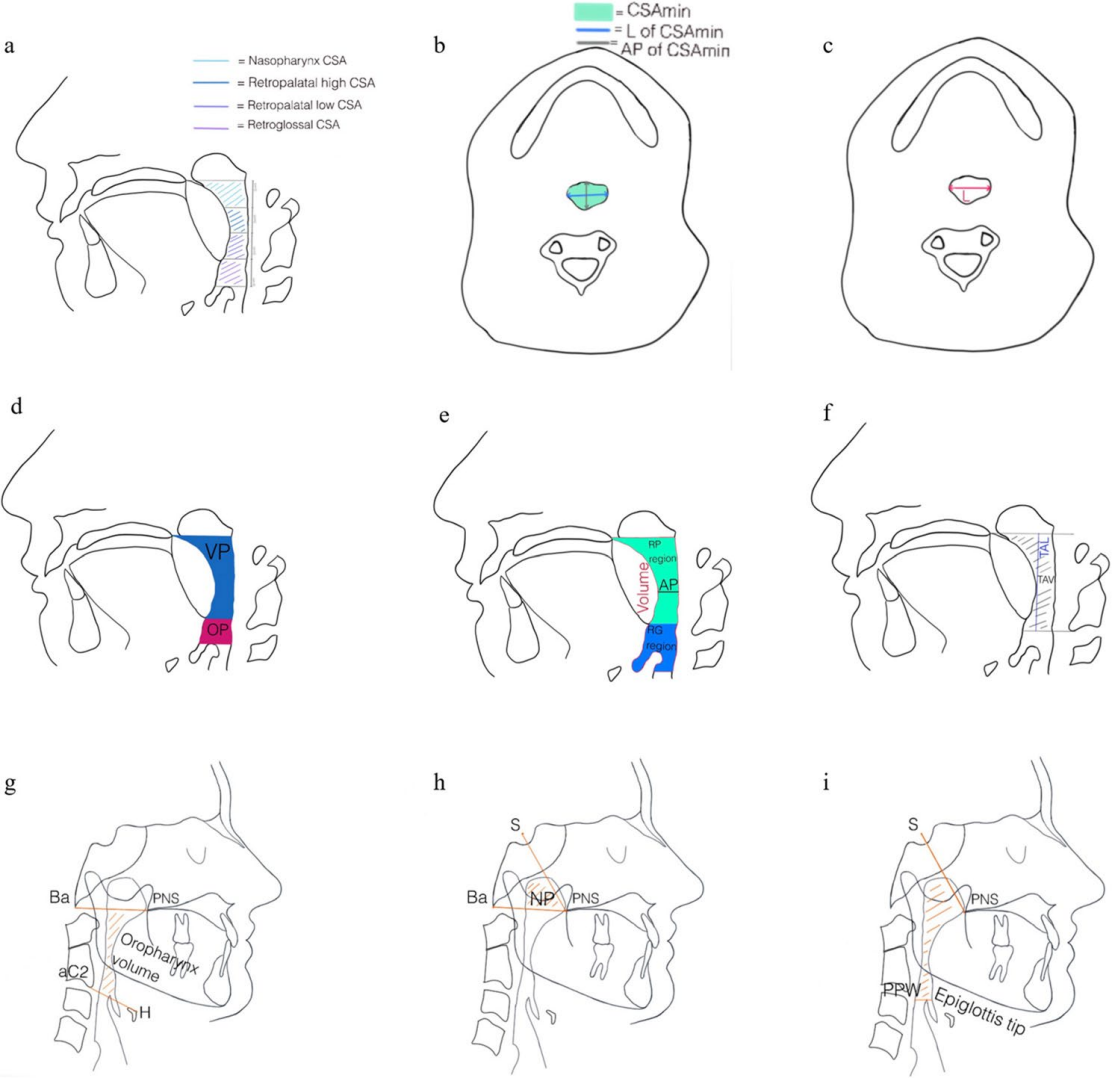
Upper airway measurements across included studies. a: cross-sectional area (CSA): CSA of the nasopharynx, high retropalatal CSA, low retropalatal CSA, retroglossal CSA [47], b: minimum cross-sectional area (CSAmin), lateral diameter of CSAmin, sagittal diameter (AP) of CSAmin [28, 29, 34, 48], c: minimum lateral diameter (L) [48], d: velopharynx (VP) and oropharynx (OP) [32], e: airway volume of the retropalatal (RP) and retroglossal (RG) region and minimum anteroposterior (AP) diameter of RP region [28, 34, 48], f: total airway volume (TAV) and total airway length (TAL) [34, 35, 48], g: oropharynx volume [29, 43], h: nasopharynx volume [38], i: nasopharynx and oropharynx volume [38]

Figure 3 presents all measurements of upper airway of the included studies. In diagnostic studies, the retropalatal region emerged as the predominant site of narrowing. While Chen et al. [28] reported significantly smaller retropalatal diameters and minimum cross-sectional area (CSAmin) in OSA, retroglossal CSAmin and total oropharyngeal airway volume did not differ between groups. Ciavarella et al. [34] reported that total airway volume, the CSAmin and the lateral diameter at CSAmin exhibited a statistically significant inverse correlation with the ODI.

In contrast, Enciso et al. [29] found significant differences only in CSAmin, lateral diameter and shape of upper airway. Median anteroposterior dimension, airway length, average cross-sectional area and total airway volume showed no significant group differences. Airway shape also did not differ significantly despite subjects with OSA presenting slightly less elliptical morphologies. Furthermore, Lam et al. [32] reported no significant differences in velopharyngeal or hypopharyngeal CSAmin across OSA severity groups or in comparison with non-OSA subjects, thereby reinforcing the inconsistency of findings outside the retropalatal region. As demonstrated by Schwab et al. [47], a statistically significant decrease in CSAmin was observed in subjects diagnosed with OSA, particularly at the retropalatal and retroglossal levels during the expiratory phase.

In intervention-dependent studies, airway expansion was consistently observed across all therapeutic modalities; however, baseline airway dimensions showed limited and inconsistent predictive value for treatment response. In RME therapy, oropharyngeal volume increased significantly both in patients with primary snoring and with OSA, yet the magnitude of enlargement did not differ statistically between both groups [36].

Chen et al. [42] revealed no baseline morphological differences between responders and non-responders to MAD therapy, nor any correlation between CSAmin and treatment outcome. Pahkala et al. [38] also reported postoperative increases in airway volume and CSAmin, regardless of clinical success. In the context of MMA surgery, postoperative airway enlargement was found to be comparable between responders and non-responders. However, responders exhibited larger baseline anteroposterior diameters and CSAmin. Nonetheless, these postoperative gains themselves did not differentiate successful from unsuccessful outcomes.

### Skeletal characteristics

Skeletal outcome measurements in CBCT and CT were highly heterogeneous across studies. Consequently, no reproducible skeletal morphology pattern could be identified across subjects with OSA.

Firincioglulari et al. [31] reported that individuals with OSA showed greater anterior mandibular thickness, reduced genial tubercle width, and increased linear distance from the lingual foramen to the mandibular base compared with subjects without OSA. In contrast, the number, frequency, and anatomical distribution of lingual foramina did not differ significantly between groups. Lam et al. [32] evaluated sagittal craniofacial dimensions, including hyoid mandibular plane distance, maxillary length and ANB angle. These craniofacial characteristics appeared to be increased in subjects with OSA compared with controls. Enciso et al. [40] reported radiological findings such as TMJ alterations or sinus variants; however, none of these differed significantly between subjects with and without OSA.

Overall, the skeletal measurements lack standardization across these included CBCT and CT with combined PG/PSG studies, and no single craniofacial measurement has been validated as a consistent discriminator between subjects with OSA, habitual snorers, and healthy controls.

**Fig. 4:**
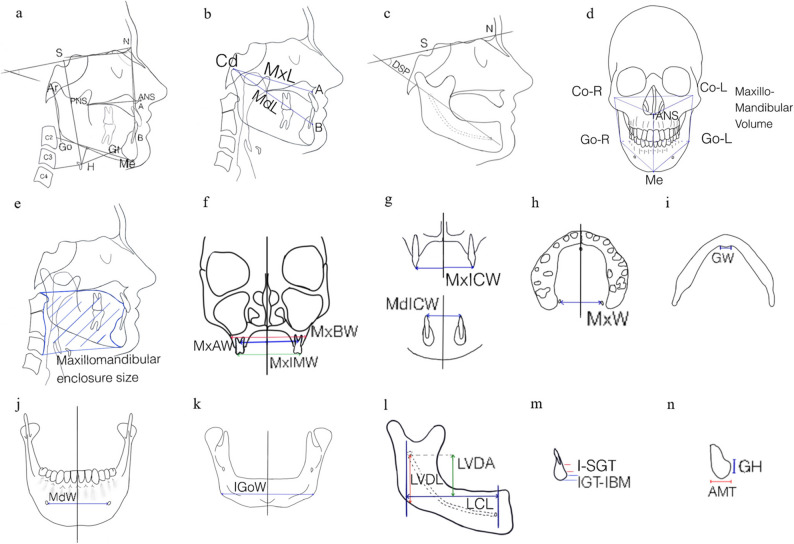
Skeletal measurements across included studies. a: sagittal relation of the maxilla to the anterior cranial base (SNA) [32], sagittal relation of the mandible to the anterior cranial base (SNB) [32], sagittal relationship between maxilla and mandible (ANB) [32], hyoid – MeGo distance (H-MeGo) [32], b: maxillary length (MxL), mandibular length (MdL) [35], c: distal segment plane angle (NS – DSP) [35], d: maxillomandibular volume [35], e: maxillomandibular enclosure size [48], f: maxillary alveolar width (MxAW), maxillary intermolar width (MxIMW), maxillary basal width (MxBW) [35], g: maxillary intercanine width (MxICW), mandibular intercanine width (MdlCW) [35], h: maxillary width [35], i: genial tubercle width [31, 35], j: mandibular width (MdW) [35], k: intergonial width (IGoW) [31], l: lingual canal length (LCL), vertical distance from lingual foramen to mandibular base (LVDL), vertical distance from lingual foramen to alveolar crest (LVDA) [31], m: lower incisor - genial tubercle distance (I-SGT), inferior genial tubercle - mandibular base distance (IGT-IBM) [31], n: genial tubercle height (GH), anterior mandibular thickness (AMT) [31]

Bariani et al. [36] reported that baseline skeletal characteristics were not predictive of volumetric airway changes or improvements in sleep variables. This indicates that craniofacial morphology derived from CT scans was not clinically informative with regard to treatment outcome. Similarly, both studies [38, 42] on MAD therapy found no significant baseline skeletal differences between responders and non-responders. Thai et al. [35] reported that patients who responded to MMA surgery had smaller preoperative SNA and SNB angles and a more cranially positioned hyoid bone. In contrast, non-responders showed a higher postoperative ANB angle (Fig. 4).

### Soft tissue

**Fig. 5:**
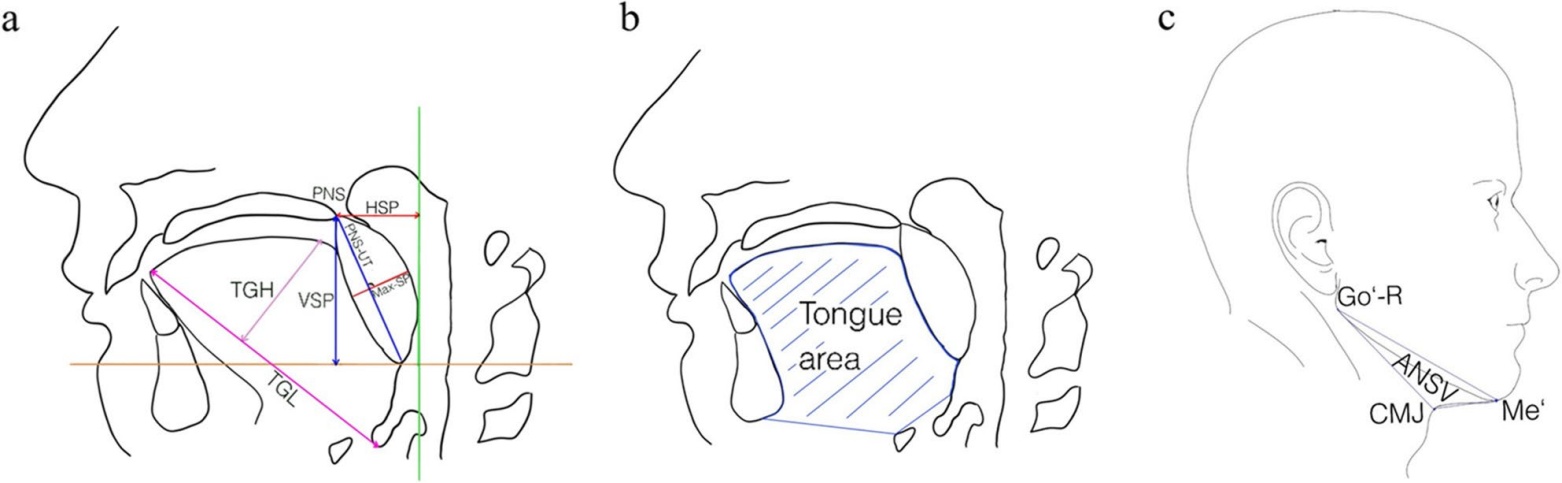
Soft tissue measurements across included studies. a: tongue length (TGL) [35], tongue height (TGH), soft palate length (PNS-UT) [32, 48], soft palate thickness (Max-Sp) [32], horizontal soft palate length (HSP) [29], and vertical soft palate length (VSP) [29], b: tongue area [48], c: anterior neck space volume (ANSV) [35]

Soft tissue assessments also showed substantial variability across studies. Lam et al. [45] found that individuals with OSA had greater vertical soft palate length and increased soft palate thickness compared with controls. However, Enciso et al. [39] found no significant differences in vertical or horizontal soft palate length between individuals with OSA and primary snorers. They also described qualitative soft tissue features, such as hypertrophic tonsils, an elongated soft palate and an enlarged tongue, that appeared to be more prevalent in individuals with moderate-to-severe OSA. However, none of these features reached statistical significance when compared with the control group [49]. In maxillomandibular advancement, responders exhibited shorter tongue and soft palate lengths than non-responders; however, soft palate thickness did not differ between groups [35] (Fig. 5).

### Influence of BMI, age and gender

In two studies, BMI was reported either for the total sample or only for the subjects with OSA.

Across studies, a higher BMI was constantly associated with greater disease severity or a reduced treatment response. For example, Chen et al. [28] found that overweight individuals (BMI ≥ 25 kg/m²) exhibited a significantly higher RDI and lower minimum oxygen saturation. These individuals also demonstrated smaller lateral dimensions and a reduced CSAmin in the retropalatal region; however, anteroposterior dimensions, retroglossal area and total oropharyngeal volume remained unchanged. Regarding treatment response Chen et al. [42] reported that responders had significantly lower baseline BMI and neck circumference than those who did not respond. Enciso et al. [29] observed that their cases of OSA had a higher BMI and larger neck circumference than primary snorers. Lam et al. [32] included subjects who differed significantly in BMI and neck circumference across the groups (no OSA, mild OSA, and severe OSA), and in their regression model, a higher BMI was consistently associated with both mild/moderate and severe OSA.

Schwab et al. [47] reported significant correlations between CSAmin and BMI, neck circumference, RDI, and oxygen saturation nadir. Correlations of similar magnitude were observed at the caudal airway levels (retropalatal-low and retroglossal), whereas no significant associations were identified at the rostral levels (nasopharynx and retropalatal-high).

One study reported age only for the total sample and not separately for the groups [34]. Overall, subjects with OSA were older than those without OSA, and age tended to increase with greater OSA severity [30–33, 49].

Enciso et al. [49] evaluated age in multivariable regression models, where it remained a significant independent predictor of OSA severity, primarily in interaction with structural airway narrowing. Similarly, Lam et al. [32] found that older age increased the risk of OSA independently of airway dimensions; however, airway dimension itself did not directly correlate with age in univariate analysis. Schwab et al. [33] reported no significant correlation between minimal airway size and age when controlling for BMI.

Regarding the effect of age on treatment response, Pahkala et al. [38] demonstrated a statistically significant age difference between responders and non-responders, with the latter being significantly older.

Most studies reported gender, but there was a marked predominance of male participants in the OSA groups [28]. Several studies included male subjects predominantly in the OSA cohorts. Some studies provided an overall gender distribution without group-specific data, while a few did not report gender at all. In contrast, the intervention-dependent studies tended to show a more balanced distribution of males and females between responders and non-responders, although overall samples remained male-dominant. No study conducted gender-stratified analyses of CBCT and CT measurements.

## Discussion

This systematic review examined whether CBCT- or CT-derived airway, craniofacial, and soft-tissue measurements provide diagnostic or prognostic value in the assessment and management of OSA. In order to address this question, the following criteria were applied to determine the inclusion of studies in the analysis: (1) the presence of PSG- or PG-confirmed non-OSA control groups, (2) the presence of clearly defined OSA severity degrees, and (3) the presence of treatment responder classifications. By implementing these rigorous methodological criteria, the present review directly evaluates the three central questions outlined in the introduction: whether CBCT and CT measurements can distinguish individuals with and without OSA, reflect disease severity, or predict treatment outcome. A comprehensive review of the included studies revealed substantial variability in anatomical definitions, imaging protocols, and measurement methods across upper-airway, skeletal, and soft-tissue. This heterogeneity, combined with the inconsistency of reporting standards, resulted in a substantial limitation of the comparability across studies and the identification of reproducible diagnostic or prognostic markers as detailed in the sections below. In addition, only a small number of studies meeting the inclusion criteria used control groups in which OSA had been formally excluded by PSG or PG; moreover, the AHI thresholds defining control groups varied considerably and OSA severity stratification was inconsistent, together precluding a meta-analysis. Individuals with OSA presented with reduced upper airway compared with non-OSA subjects and primary snorers. Despite different scan protocols and measurement definitions, the retropalatal region was the predominant site of airway narrowing; however, this finding alone is insufficient for diagnosis or severity grading. Besides local airway dimensions, functional factors and craniofacial morphology contribute substantially to disease development. Craniofacial characteristics that can be assessed in lateral cephalometric analysis (e.g., pronounced mandibular retrognathia, vertical growth pattern, or maxillary hypoplasia) as well as factors such as body weight and age play an important role. As these variables were insufficiently controlled for in most included studies, the isolated interpretation of retropalatal narrowing is clinically limited. Furthermore, imaging is performed in awake patients and therefore does not reflect the dynamic sleep-related airway collapsibility or the temporal occurrence of apneic events.

Most studies reported smaller anteroposterior and lateral diameters of CSAmin in individuals with OSA, whereas associations with total airway volume or retroglossal dimensions were inconsistent. Several studies found significant correlations between CSAmin and OSA diagnosis or ODI, while others reported no differences in volumetric or dimensional variables. Overall, only a limited subset of measurements - primarily retropalatal CSAmin - demonstrated reproducible group differences. In intervention-dependent studies, all therapeutic modalities resulted in upper airway enlargement; however, baseline dimensions showed limited value in predicting treatment response. Airway expansion following RME, MAD therapy, or MMA occurred regardless of clinical outcome. Although responders to MMA exhibited larger baseline oropharyngeal diameters and CSAmin, postoperative gains were comparable between responders and non-responders. Collectively, these findings indicate that pretreatment airway morphology offers restricted prognostic utility, while treatment-induced airway changes are consistent but not directly linked to therapeutic success. Bariani et al. [43] concluded that volumetric airway changes after RME should not be considered a valid or reliable indicator of breathing improvement in children, as most patients exhibit volumetric increases that do not correspond to measurable respiratory benefit. Based on their findings, Chen et al. [44] concluded that there is currently no justification for routinely performing CBCT in patients with OSA to determine their response to MAD therapy.

Recent analyses have shown that, although postoperative airway enlargement may partially relapse, OSA improvement remains stable. Navasumrit et al. [50] reported that OSA improvement after MMA remained stable despite the relapse of the upper airway. These findings suggest that changes in static airway caliber may not fully explain clinical improvement [14, 51]. Postoperative improvement has been reported alongside skeletal and soft-tissue positional changes (e.g., maxillary and tongue displacement), although causal mechanisms cannot be inferred from the available evidence [50]. Taken together, these findings indicate that pretreatment airway morphology showed inconsistent associations with treatment outcome and that treatment-induced dimensional changes, although consistently present, does not allow reliable prediction.

The skeletal findings across the included studies showed considerable heterogeneity in measurement parameters and definitions. Thus, some studies showed differences between individuals with OSA and without OSA such as alterations in mandibular cortical dimensions, variations in sagittal jaw relationships, or differences in hyoid position. In non-surgical interventions, particularly RME and MAD therapy, baseline skeletal dimensions were unable to distinguish responders from non-responders, while responders to MMA presented with shorter maxillary length and a more superior-anterior hyoid position than non-responders. In this context, it is noteworthy that for the assessment of skeletal characteristics in non-surgical treatment planning, a lateral cephalogram often provides sufficient diagnostic information. Cephalometry provides an accepted and standardized method for the evaluation of sagittal jaw relationships, vertical growth pattern and hyoid position, without the additional radiation exposure associated with CBCT and CT.

Several studies reported an elongated soft-palate morphology in patients with OSA, whereas others found no significant differences in comparison with controls or primary snorers. Qualitative observations, including hypertrophic tonsils, an elongated velum, and an enlarged tongue, were documented more frequently in moderate to severe OSA. However, these findings did not attain statistical significance. Furthermore, the results of prognostic assessments were found to be inconsistent. In patients undergoing MMA, responders were reported to exhibit shorter tongues and shorter soft palates.

However, due to substantial methodological heterogeneity across studies assessing soft-tissue parameters, no robust evidence can be derived to establish standardized predictive markers. Furthermore, soft tissue is inherently dynamic, dependent on volume, respiratory phase, and body position - factors that cannot be captured in static CBCT or CT imaging. Consequently, it can be hypothesized that soft-tissue measurements may have limited ability to differentiate groups or to relate consistently to clinical outcomes.

### Limitation and strengths

This systematic review has several notable strengths. Most importantly, it applied strict inclusion criteria requiring that OSA diagnoses - and, critically, the absence of OSA in control participants - be objectively confirmed by PSG or PG. This requirement is essential, as the prevalence of unrecognized OSA is high even among ostensibly healthy individuals. By enforcing this standard, the review minimizes misclassification bias. Many studies were excluded because their control groups were not verified using PG or PSG, and because OSA severity categories were not clearly defined. Several studies also failed to report PSG or PG parameters in sufficient detail. For example, Sadry et al. [52] provided no information on sleep-related variables such as AHI, RDI, or ODI. Instead, individuals were simply described as being divided into a “healthy control group“ and an “OSA group”, without specifying diagnostic thresholds or OSA severity degrees.

Consequently, only a small number of studies met all the inclusion criteria. Of these, only 11 provided data in which OSA and non-OSA was diagnosed using PSG or PG.

Most of the included studies exhibited a high risk of bias. While some systematic reviews exclude studies with high bias scores, the QUIPS tool was used to demonstrate the overall quality of the research in this area and emphasize that there is currently insufficient evidence to support the relevance of CBCT and CT parameters.

A major limitation of the included studies was the presence of key confounding variables – BMI, neck circumference, age, and gender – which substantially influence the risk of OSA. Individuals with OSA were consistently older and had a higher BMI than the control groups and were also more frequently male. As these variables are established risk factors for OSA, they act as major confounders [53–55]. As most studies did not adjust for age, BMI or gender, any observed group differences in airway measurements, such as reduced posterior airway space, cannot be attributed to OSA itself.

Higher BMI increases parapharyngeal fat deposition, advancing age reduces upper airway muscle tone and male sex is associated with distinct fat distribution and craniofacial patterns. Each of these factors can independently reduce airway dimensions. When baseline characteristics differ substantially and no statistical adjustment is applied, observed CBCT differences may reflect these underlying disparities rather than disease-specific anatomical traits.

The included studies also demonstrated substantial methodological heterogeneity. Only three studies used an AHI-based severity classification consistent with AASM guidelines, defining non-OSA as an AHI of less than 5.

Other studies relied on alternative criteria, such as RDI-based classifications or thresholds that categorized individuals with primary snoring as non-OSA with an AHI < 10. Comparable variability was observed among intervention-dependent studies with respect to definitions of treatment response: Chen et al. [42] classified responders as those with post-treatment AHI < 10 and > 50% reduction; Pahkala et al. [46] distinguished complete responders (AHI ≤ 5) from partial and non-responders using mixed criteria; and Thai et al. [35] employed a threshold of post-operative AHI < 10 and > 50% reduction. Furthermore, only one study [43] investigating pediatric patients, two studies [44, 46] evaluating MAD therapy, and one study [35] examining surgical intervention met the inclusion criteria.

This degree of heterogeneity limited the ability to perform meaningful comparisons or meta-analytic synthesis and underscores the lack of standardized study designs and outcome definitions within this field.

Moreover, scanning positions varied considerably in terms of body position. Most diagnostic CT and CBCT data sets were acquired in the supine position, while several therapeutic CBCT scans were performed in the upright position. However, since none of the studies provided paired measurements in the upright and supine positions in the same individuals, a direct position effect cannot be quantified. Furthermore, the acquisition of such paired measurements would not be ethically justifiable.

Another important factor is the influence of respiration on airway dimensions. Only two [28, 56] of the 11 studies provided respiratory instructions to participants. Chen et al. [28] instructed subjects to exhale during image acquisition, whereas Schwab et al. [47] synchronized CT slice acquisition with respiratory signals and demonstrated that airway caliber is significantly affected by the respiratory cycle, reaching its minimum at the end of expiration in both OSA and non-OSA individuals. This highlights the significant impact of the respiratory phase on airway measurements.

Furthermore, the lack of standardized imaging protocols and the combination of manual and software-assisted methods or qualitative assessments resulted in limited methodological comparability across studies. In most studies, CBCT and CT measurements were also not performed under blinded conditions. This raises the possibility that examiners were aware of group allocation, introducing substantial potential for systematic measurement error. Knowledge of whether a participant belonged to the OSA or control group, responder or non-responder group could influence the identification of landmarks. The lack of blinding therefore represents a major methodological limitation across the included studies, substantially decreasing confidence in the validity of the reported associations between CBCT-derived airway measures and OSA severity.

### Clinical implications

Based on the available evidence, CBCT and CT do not provide clinically meaningful diagnostic or prognostic information for the assessment or management of OSA in dental practice. Airway measurements neither allow reliable identification of OSA nor differentiate disease severity, nor do they predict treatment response.

As repeatedly highlighted in the literature, a fundamental limitation of CBCT and CT is that these scans capture only a brief static snapshot of the airway in an awake individual, typically during either inspiration or expiration.

Because upper-airway patency during sleep is a dynamic phenomenon influenced by neuromuscular tone and respiratory effort, static imaging obtained while awake cannot reflect the pathophysiological processes underlying OSA. Consequently, current evidence does not support a diagnostic or therapeutic value of CBCT- or CT-based airway assessment, and it should therefore not be used to guide clinical decision-making in dental sleep medicine.

Given the substantial radiation exposure associated with CT and CBCT, there needs to be considerable clinical benefit to justify their use. Given the limitations of the available evidence, routine airway assessment using CBCT or CT is not supported for OSA-related purposes.

In routine dental and orthodontic settings, OSA screening should instead focus on clinical risk assessment. Dentists and orthodontists must be able to recognize signs and symptoms suggestive of OSA and obtain a comprehensive medical history that addresses established risk factors, including prior OSA diagnosis, daytime sleepiness, loud snoring, witnessed apneas, hypertension, increased BMI, neck circumference and age, gender and relevant comorbidities. In adults, validated screening instruments, particularly the ESS and STOP-BANG questionnaire, provide an effective method of identifying individuals at low, intermediate or high risk of OSA.

Individuals screening positive should be referred to a sleep physician for definitive evaluation using PSG or PG. Radiographic assessment of airway size or morphology cannot replace this process, as imaging findings do not correlate reliably with PSG outcomes and therefore should be interpreted cautiously.

Three-dimensional imaging remains necessary in specific surgical contexts, particularly for orthognathic planning or orthodontic treatment planning in accordance with established guidelines [57]. In such cases, the indication for imaging is determined by the underlying craniofacial diagnosis rather than by the assessment of a suspected OSA.

Intervention-related evidence supports this distinction. Although various treatments may enlarge the upper airway, these volumetric changes do not correspond to measurable improvements in polysomnographic outcomes. Furthermore, responders and non-responders cannot be reliably differentiated using CBCT-derived airway parameters, and predictors of therapeutic success are related primarily to craniofacial morphology rather than airway volume itself. Consequently, treatment outcome should be evaluated by repeat PG or PSG performed by a sleep physician.

When three-dimensional imaging has been obtained for justified orthodontic or surgical indications, the airway and surrounding structures may be assessed as part of the existing dataset and interpreted together with clinical findings. However, current evidence does not support acquiring CBCT or CT solely for OSA screening or for prediction of treatment response [11, 22].

## Conclusion

This systematic review addresses the evidence gap identified by the AAOMR in 2011 by showing that the available evidence does not support clinically meaningful diagnostic or prognostic value of CBCT or CT for assessing OSA.

The limited number of eligible studies had highly heterogeneous upper-airway, craniofacial, and soft-tissue measurements that lacked common patterns. While individuals with OSA often exhibited smaller retropalatal dimensions, particularly reduced CSAmin, the findings regarding the retroglossal airway, total airway volume, skeletal morphology, and soft tissue structures were inconsistent and not associated with disease severity and thus did not adequately represent the clinical expression of OSA.

Most of the included studies involved OSA cohorts with substantially higher BMI, older age, and greater proportions of males, and few studies adjusted for these critical confounders, which themselves are highly associated with higher OSA risk. Consequently, anatomical differences – whether airway, skeletal, or soft tissue – cannot be attributed to OSA itself. Furthermore, CBCT and CT-derived measurements did not predict treatment response in orthodontic, surgical, or oral-appliance interventions, and postoperative airway enlargement did not correlate with improvements in PSG outcomes.

Overall, the available evidence suggests that CBCT- or CT-based assessments of the upper airway, craniofacial structures, and soft tissues do not contribute to the diagnosis, grading, or management of OSA. Accordingly, CBCT should not be used for OSA screening or treatment prediction and should only be performed when clinically justified according to established dental guidelines or when required for surgical treatment planning.

## Supplementary Information

Supplementary Material 1.

## Data Availability

No datasets were generated or analysed during the current study.

## References

[1] Gottlieb DJ, Punjabi NM. Diagnosis and Management of Obstructive Sleep Apnea: A Review. JAMA. 2020;323:1389. 10.1001/jama.2020.3514. 32286648 10.1001/jama.2020.3514

[2] Marin JM, Carrizo SJ, Vicente E, Agusti AGN. Long-term cardiovascular outcomes in men with obstructive sleep apnoea-hypopnoea with or without treatment with continuous positive airway pressure: an observational study. Lancet. 2005;365:1046–53. 10.1016/S0140-6736(05)71141-7. 15781100 10.1016/S0140-6736(05)71141-7

[3] Vaienti B, Di Blasio M, Arcidiacono L, Santagostini A, Di Blasio A, Segù M. A narrative review on obstructive sleep apnoea syndrome in paediatric population. Front Neurol. 2024;15:1393272. 10.3389/fneur.2024.1393272. 39036631 10.3389/fneur.2024.1393272 PMC11257894

[4] Benjafield AV, Ayas NT, Eastwood PR, Heinzer R, Ip MSM, Morrell MJ, et al. Estimation of the global prevalence and burden of obstructive sleep apnoea: a literature-based analysis. Lancet Respir Med. 2019;7:687–98. 10.1016/S2213-2600(19)30198-5. 31300334 10.1016/S2213-2600(19)30198-5 PMC7007763

[5] Kapur VK, Auckley DH, Chowdhuri S, Kuhlmann DC, Mehra R, Ramar K, et al. Clinical Practice Guideline for Diagnostic Testing for Adult Obstructive Sleep Apnea: An American Academy of Sleep Medicine Clinical Practice Guideline. J Clin Sleep Med. 2017;13:479–504. 10.5664/jcsm.6506. 28162150 10.5664/jcsm.6506 PMC5337595

[6] American Academy of Sleep Medicine. editor. International Classification of Sleep Disorders, (ICSD-3). 3rd ed. Darien, IL American Academy of Sleep Medicine; 2014.

[7] Johns MW. A New Method for Measuring Daytime Sleepiness: The Epworth Sleepiness Scale. Sleep. 1991;14:540–5. 10.1093/sleep/14.6.540. 1798888 10.1093/sleep/14.6.540

[8] Chung F, Abdullah HR, Liao P, STOP-Bang Questionnaire. Chest. 2016;149:631–8. 10.1378/chest.15-0903. 26378880 10.1378/chest.15-0903

[9] Finke H, Drews A, Engel C, Koos B. Craniofacial risk factors for obstructive sleep apnea—systematic review and meta-analysis. J Sleep Res. 2024;33:e14004. 10.1111/jsr.14004. 37485571 10.1111/jsr.14004

[10] Eckert DJ. Phenotypic approaches to obstructive sleep apnoea – New pathways for targeted therapy. Sleep Med Rev. 2018;37:45–59. 10.1016/j.smrv.2016.12.003. 28110857 10.1016/j.smrv.2016.12.003

[11] Behrents RG, Shelgikar AV, Conley RS, Flores-Mir C, Hans M, Levine M, et al. Obstructive sleep apnea and orthodontics: An American Association of Orthodontists White Paper. Am J Orthod Dentofac Orthop. 2019;156:13–e281. 10.1016/j.ajodo.2019.04.009. 10.1016/j.ajodo.2019.04.009 31256826

[12] Epstein. Clinical Guideline for the Evaluation, Management and Long-term Care of Obstructive Sleep Apnea in Adults. J Clin Sleep Med. 2009;05:263–76. 10.5664/jcsm.27497. PMC2699173 19960649

[13] Zaghi S, Holty J-EC, Certal V, Abdullatif J, Guilleminault C, Powell NB, et al. Maxillomandibular Advancement for Treatment of Obstructive Sleep Apnea: A Meta-analysis. JAMA Otolaryngol Neck Surg. 2016;142:58. 10.1001/jamaoto.2015.2678. 10.1001/jamaoto.2015.2678 26606321

[14] Hsieh Y-J, Liao Y-F, Chen N-H, Chen Y-R. Changes in the calibre of the upper airway and the surrounding structures after maxillomandibular advancement for obstructive sleep apnoea. Br J Oral Maxillofac Surg. 2014;52:445–51. 10.1016/j.bjoms.2014.02.006. 24629456 10.1016/j.bjoms.2014.02.006

[15] Randerath W, Verbraecken J, De Raaff CAL, Hedner J, Herkenrath S, Hohenhorst W, et al. European Respiratory Society guideline on non-CPAP therapies for obstructive sleep apnoea. Eur Respir Rev. 2021;30:210200. 10.1183/16000617.0200-2021. 34853097 10.1183/16000617.0200-2021 PMC9489103

[16] Ramar K, Dort LC, Katz SG, Lettieri CJ, Harrod CG, Thomas SM, et al. Clinical Practice Guideline for the Treatment of Obstructive Sleep Apnea and Snoring with Oral Appliance Therapy: An Update for 2015: An American Academy of Sleep Medicine and American Academy of Dental Sleep Medicine Clinical Practice Guideline. J Clin Sleep Med. 2015;11:773–827. 10.5664/jcsm.4858. 26094920 10.5664/jcsm.4858 PMC4481062

[17] Pereira A, Gurgel M, Pereira R, Fabbro CD, de Barros Silva P, Costa F, et al. Evaluation of condylar and mandibular movements on the upper airway during the use of mandibular advancement device for obstructive sleep apnea treatment. Clin Oral Investig. 2024;28:122. 10.1007/s00784-024-05513-9. 38286954 10.1007/s00784-024-05513-9 PMC11066818

[18] Venza N, Malara A, Liguori C, Cozza P, Laganà G. Upper Airway Characteristics and Morphological Changes by Different MADs in OSA Adult Subjects Assessed by CBCT 3D Imaging. J Clin Med. 2023;12:5315. 10.3390/jcm12165315. 37629359 10.3390/jcm12165315 PMC10455815

[19] Chen H, Eckert DJ, Van Der Stelt PF, Guo J, Ge S, Emami E, et al. Phenotypes of responders to mandibular advancement device therapy in obstructive sleep apnea patients: A systematic review and meta-analysis. Sleep Med Rev. 2020;49:101229. 10.1016/j.smrv.2019.101229. 31785583 10.1016/j.smrv.2019.101229

[20] Alessandri-Bonetti A, Bortolotti F, Moreno-Hay I, Michelotti A, Cordaro M, Alessandri-Bonetti G, et al. Effects of mandibular advancement device for obstructive sleep apnea on temporomandibular disorders: A systematic review and meta-analysis. Sleep Med Rev. 2019;48:101211. 10.1016/j.smrv.2019.101211. 31605905 10.1016/j.smrv.2019.101211

[21] Gurgel ML, Junior CC, Cevidanes LHS, De Barros Silva PG, Carvalho FSR, Kurita LM, et al. Methodological parameters for upper airway assessment by cone-beam computed tomography in adults with obstructive sleep apnea: a systematic review of the literature and meta-analysis. Sleep Breath. 2023;27:1–30. 10.1007/s11325-022-02582-6. 35190957 10.1007/s11325-022-02582-6 PMC9392812

[22] American Academy of Oral and Maxillofacial Radiology. Clinical recommendations regarding use of cone beam computed tomography in orthodontics. Position statement by the American Academy of Oral and Maxillofacial Radiology. Oral Surg Oral Med Oral Pathol Oral Radiol. 2013;116:238–57. 10.1016/j.oooo.2013.06.002. 23849378 10.1016/j.oooo.2013.06.002

[23] Wei Z, Zhao T, Li Y, Ngan P, Wang Z, Hua F, et al. The dentofacial and upper airway morphology of adults with obstructive sleep apnea: A systematic review and meta-analysis. Sleep Med Rev. 2025;80:102065. 10.1016/j.smrv.2025.102065. 39899914 10.1016/j.smrv.2025.102065

[24] Kazmouz S, Calzadilla N, Choudhary A, McGinn LS, Seaman A, Purnell CA. Radiographic findings predictive of obstructive sleep apnea in adults: A systematic review and meta-analysis. J Cranio-Maxillofac Surg. 2025;53:162–80. 10.1016/j.jcms.2024.11.003. 10.1016/j.jcms.2024.11.003 39609122

[25] Taha YM, Abu El Sadat SM, Gaber RM, Farid MM. Ability of upper airway metrics to predict obstructive sleep apnea severity: a systematic review. Dentomaxillofacial Radiol. 2025;54:245–55. 10.1093/dmfr/twaf010. 10.1093/dmfr/twaf010 39903052

[26] PRISMA statement. PRISMA statement. https://www.prisma-statement.org. Accessed 2 Sept 2025.

[27] Hayden JA, Côté P, Bombardier C. Evaluation of the Quality of Prognosis Studies in Systematic Reviews. Ann Intern Med. 2006;144:427–37. 10.7326/0003-4819-144-6-200603210-00010. 16549855 10.7326/0003-4819-144-6-200603210-00010

[28] Chen N, Li KK, Li S, Wong C, Chuang M, Hwang C, et al. Airway Assessment by Volumetric Computed Tomography in Snorers and Subjects With Obstructive Sleep Apnea in a Far-East Asian Population (Chinese). Laryngoscope. 2002;112:721–6. 10.1097/00005537-200204000-00023. 12150529 10.1097/00005537-200204000-00023

[29] Enciso R, Nguyen M, Shigeta Y, Ogawa T, Clark GT. Comparison of CBCT parameters and sleep questionnaires in sleep questionnaires in sleep apnea patients and controls. Oral Surg Oral Med Oral Pathol Oral Radiol Endod. 2011;109:285–93. 10.1016/j.tripleo.2009.09.033. 10.1016/j.tripleo.2009.09.033 PMC2900776 20123412

[30] Enciso R, Shigeta Y, Nguyen M, Clark GT. Comparison of Cone-Beam CT Incidental Findings between Moderate/Severe Obstructive Sleep Apnea patients and Mild/ Normal patients. Oral Surg Oral Med Oral Pathol Oral Radiol. 2013;114:373–81. 10.1016/j.oooo.2012.03.014. 10.1016/j.oooo.2012.03.014 PMC3428237 22862979

[31] Firincioglulari M, Aksoy S, Orhan K, Oz U, Rasmussen F. Comparison of anterior mandible anatomical characteristics between obstructive sleep apnea patients and healthy individuals: a combined cone beam computed tomography and polysomnographic study. Eur Arch Otorhinolaryngol. 2020;277:1427–36. 10.1007/s00405-020-05805-2. 31980885 10.1007/s00405-020-05805-2

[32] Lam B, Ooi CGC, Peh WCG, Lauder I, Tsang KWT, Lam W-K, et al. Computed tomographic evaluation of the role of craniofacial and upper airway morphology in obstructive sleep apnea in Chinese. Respir Med. 2004;98:301–7. 10.1016/j.rmed.2003.10.009. 15072170 10.1016/j.rmed.2003.10.009

[33] Schwab RJ, Gefter WB, Hoffman EA, Gupta KB, Pack AI. Dynamic Upper Airway Imaging during Awake Respiration in Normal Subjects and Patients with Sleep Disordered Breathing. Am Rev Respir Dis. 1993;148:1385–400. 10.1164/ajrccm/148.5.1385. 8239180 10.1164/ajrccm/148.5.1385

[34] Ciavarella D, Ferrara D, Spinoso G, Cattaneo P, Leo C, Russo LL, et al. Airway Analysis and Morphometric Assessment of Dental Arches in Obstructive Sleep Apnea Patients. J Clin Med. 2025;14:296. 10.3390/jcm14020296. 39860302 10.3390/jcm14020296 PMC11766405

[35] Thai KH, Chen Y-A, Yao C-F, Chen N-H, Liao Y-F, Chen Y-R. Comparison of phenotypic characteristics between responders and non-responders to treatment for obstructive sleep apnea with maxillomandibular advancement in adults. Sleep Med. 2025;129:346–53. 10.1016/j.sleep.2025.03.006. 40101536 10.1016/j.sleep.2025.03.006

[36] Bariani RCB, Bigliazzi R, Badreddine FR, Yamamoto LH, Tufik S, Moreira G, et al. A clinical trial on 3D CT scan and polysomnographyc changes after rapid maxillary expansion in children with snoring. Braz J Otorhinolaryngol. 2022;88:S162–70. 10.1016/j.bjorl.2022.04.004. 35780010 10.1016/j.bjorl.2022.04.004 PMC9801059

[37] Chen H, Aarab G, Lobbezoo F, De Lange J, Van der Stelt P, Darendeliler MA, et al. Differences in three-dimensional craniofacial anatomy between responders and non-responders to mandibular advancement splint treatment in obstructive sleep apnoea patients. Eur J Orthod. 2019;41:308–15. 10.1093/ejo/cjy085. 30624726 10.1093/ejo/cjy085

[38] Pahkala R, Seppä J, Myllykangas R, Tervaniemi J, Vartiainen VM, Suominen AL, et al. The impact of oral appliance therapy with moderate mandibular advancement on obstructive sleep apnea and upper airway volume. Sleep Breath. 2020;24:865–73. 10.1007/s11325-019-01914-3. 31401736 10.1007/s11325-019-01914-3 PMC7426308

[39] Enciso R, Nguyen M, Shigeta Y, Ogawa T, Clark GT. Comparison of cone-beam CT parameters and sleep questionnaires in sleep apnea patients and control subjects. Oral Surg Oral Med Oral Pathol Oral Radiol Endodontology. 2010;109:285–93. 10.1016/j.tripleo.2009.09.033. 10.1016/j.tripleo.2009.09.033 PMC2900776 20123412

[40] Enciso R, Shigeta Y, Nguyen M, Clark GT. Comparison of Cone-Beam CT Incidental Findings between Moderate/Severe Obstructive Sleep Apnea patients and Mild/Normal patients. Oral Surg Oral Med Oral Pathol Oral Radiol. 2012;114:373–81. 10.1016/j.oooo.2012.03.014. 22862979 10.1016/j.oooo.2012.03.014 PMC3428237

[41] Firincioglulari M, Aksoy S, Orhan K, Oz U, Rasmussen F. Comparison of anterior mandible anatomical characteristics between obstructive sleep apnea patients and healthy individuals: a combined cone beam computed tomography and polysomnographic study. Eur Arch Oto-Rhino-Laryngol Off J Eur Fed Oto-Rhino-Laryngol Soc EUFOS Affil Ger Soc Oto-Rhino-Laryngol -. Head Neck Surg. 2020;277:1427–36. 10.1007/s00405-020-05805-2. 10.1007/s00405-020-05805-2 31980885

[42] Chen H, Aarab G, Lobbezoo F, De Lange J, Van Der Stelt P, Darendeliler MA, et al. Differences in three-dimensional craniofacial anatomy between responders and non-responders to mandibular advancement splint treatment in obstructive sleep apnoea patients. Eur J Orthod. 2019;41:308–15. 10.1093/ejo/cjy085. 30624726 10.1093/ejo/cjy085

[43] Bariani RCB, Bigliazzi R, Badreddine FR, Yamamoto LH, Tufik S, Moreira G, et al. A clinical trial on 3D CT scan and polysomnographyc changes after rapid maxillary expansion in children with snoring. Braz J Otorhinolaryngol. 2022;88(5 Suppl 5):S162–70. 10.1016/j.bjorl.2022.04.004. 35780010 10.1016/j.bjorl.2022.04.004 PMC9801059

[44] Chen H, Aarab G, Lobbezoo F, De Lange J, Van der Stelt P, Darendeliler MA, et al. Differences in three-dimensional craniofacial anatomy between responders and non-responders to mandibular advancement splint treatment in obstructive sleep apnoea patients. Eur J Orthod. 2019;41:308–15. 10.1093/ejo/cjy085. 30624726 10.1093/ejo/cjy085

[45] Lam B, Ooi CGC, Peh WCG, Lauder I, Tsang KWT, Lam W-K, et al. Computed tomographic evaluation of the role of craniofacial and upper airway morphology in obstructive sleep apnea in Chinese. Respir Med. 2004;98:301–7. 10.1016/j.rmed.2003.10.009. 15072170 10.1016/j.rmed.2003.10.009

[46] Pahkala R, Seppä J, Myllykangas R, Tervaniemi J, Vartiainen VM, Suominen AL, et al. The impact of oral appliance therapy with moderate mandibular advancement on obstructive sleep apnea and upper airway volume. Sleep Breath Schlaf Atm. 2020;24:865–73. 10.1007/s11325-019-01914-3. 10.1007/s11325-019-01914-3 PMC7426308 31401736

[47] Schwab RJ, Gefter WB, Hoffman EA, Gupta KB, Pack AI. Dynamic Upper Airway Imaging during Awake Respiration in Normal Subjects and Patients with Sleep Disordered Breathing. Am Rev Respir Dis. 1993;148:1385–400. 10.1164/ajrccm/148.5.1385. 8239180 10.1164/ajrccm/148.5.1385

[48] Chen H, Aarab G, Lobbezoo F, De Lange J, Van Der Stelt P, Darendeliler MA, et al. Differences in three-dimensional craniofacial anatomy between responders and non-responders to mandibular advancement splint treatment in obstructive sleep apnoea patients. Eur J Orthod. 2019;41:308–15. 10.1093/ejo/cjy085. 30624726 10.1093/ejo/cjy085

[49] Enciso R, Nguyen M, Clark GT. Comparison of cone beam CT incidental findings between obstructive sleep apnea patients and snorers. Sleep Breath. 2011;15:253–4. 10.1007/s11325-011-0509-x.

[50] Navasumrit S, Chen Y-A, Hsieh Y-J, Yao C-F, Chang C-S, Chen N-H, et al. Skeletal and upper airway stability following modified maxillomandibular advancement for treatment of obstructive sleep apnea in skeletal class I or II deformity. Clin Oral Investig. 2022;26:3239–50. 10.1007/s00784-021-04306-8. 35088225 10.1007/s00784-021-04306-8

[51] Susarla SM, Abramson ZR, Dodson TB, Kaban LB. Upper Airway Length Decreases After Maxillomandibular Advancement in Patients With Obstructive Sleep Apnea. J Oral Maxillofac Surg. 2011;69:2872–8. 10.1016/j.joms.2011.01.005. 21507540 10.1016/j.joms.2011.01.005

[52] Sadry S, Koca CG, Kurtulmuş H. Cone beam computed tomography analysis results in patients with obstructive sleep apnoea syndrome. Int J Clin Pract. 2021;75. 10.1111/ijcp.14497. 10.1111/ijcp.14497 34236117

[53] Tufik S, Santos-Silva R, Taddei JA, Bittencourt LRA. Obstructive Sleep Apnea Syndrome in the Sao Paulo Epidemiologic Sleep Study. Sleep Med. 2010;11:441–6. 10.1016/j.sleep.2009.10.005. 20362502 10.1016/j.sleep.2009.10.005

[54] Senaratna CV, Perret JL, Lodge CJ, Lowe AJ, Campbell BE, Matheson MC, et al. Prevalence of obstructive sleep apnea in the general population: A systematic review. Sleep Med Rev. 2017;34:70–81. 10.1016/j.smrv.2016.07.002. 27568340 10.1016/j.smrv.2016.07.002

[55] Chung F, Vairavanathan S. A Tool to Screen Patients for Obstructive. Sleep Apnea. 2008;108:10. 10.1097/ALN.0b013e31816d83e4 18431116

[56] Schwab RJ, Gupta KB, Gefter WB, Metzger LJ, Hoffman EA, Pack AI. Upper airway and soft tissue anatomy in normal subjects and patients with sleep-disordered breathing. Significance of the lateral pharyngeal walls. Am J Respir Crit Care Med. 1995;152:1673–89. 10.1164/ajrccm.152.5.7582313. 7582313 10.1164/ajrccm.152.5.7582313

[57] ARö, DGZMK: Dentale digitale Volumentomographie, Langversion 3.0. AWMF Registernummer. 2022:083 – 005. html, Zugriff am: 08.11.2025 https://www.awmf.org/leitlinien/detail/II/083-005.

