# Supplementary material for: Diagnostic and prognostic value of (cone-beam) computed tomography in dental sleep medicine for obstructive sleep apnea: a systematic review
Source: Head Face Med. 2026 Mar 12;22:36. doi: 10.1186/s13005-026-00609-x (PMC13063646; doi:10.1186/s13005-026-00609-x)
Supplement: Supplementary file 1 — Supplementary Material 1. [file 13005_2026_609_MOESM1_ESM.docx]

**Appendix I** Results of the database search conducted on September 01, 2025.

PubMed (MEDLINE including MEDLINE In-Process) (1948 to September 01, 2025)

| Query | Results |
| --- | --- |
| (Obstructive sleep apnea*[tiab] OR Obstructive sleep apnoea*[tiab] OR OSA[tiab] OR OSAHS[tiab] OR SRBD[tiab] OR sleep related breathing disorder[tiab] OR sleep-disordered breathing[tiab] OR SDB[tiab] OR Sleep Apnea Hypopnea*[tiab] OR Sleep Apnoea Hypopnoea*[tiab] OR Upper Airway Resistance Sleep Apnea*[tiab] OR Upper Airway Resistance Sleep Apnoea*[tiab] OR Sleep Apnea, Obstructive[Mesh]) AND (Dentistry[Mesh] OR Dentists[Mesh] OR dentist*[tiab] OR dental*[tiab]) AND (Cone-Beam Computed Tomography[Mesh] OR cone-beam[tiab] OR CBCT[tiab] OR tomography, x ray computed[Mesh] OR tomography, x ray computed[tiab]) NOT (case report*[ti] OR case reports[pt]) AND (German[la] OR English[la]) NOT (animals[Mesh] NOT humans[Mesh]) | 201 |

Embase.com (Elsevier) (1974 to September 01, 2025)

| Query | Results |
| --- | --- |
| ('obstructive sleep apnea'/exp OR 'Obstructive sleep apnea*':ab,ti,kw OR 'Obstructive sleep apnoea*':ab,ti,  kw OR 'OSA':ab,ti,kw OR 'OSAHS':ab,ti,kw OR 'SRBD':ab,ti,kw OR 'sleep related breathing disorder':  ab,ti,kw OR 'sleep-disordered breathing':ab,ti,kw OR 'SDB':ab,ti,kw OR 'Sleep Apnea Hypopnea*':ab,ti,kw  OR 'Sleep Apnoea Hypopnoea*':ab,ti,kw OR 'Upper Airway Resistance Sleep Apnea*':ab,ti,kw OR  'Upper Airway Resistance Sleep Apnoea*':ab,ti,kw) AND ('Dentistry'/exp OR 'Dentist'/exp OR 'dentist*':  ab,ti,kw OR 'dental*':ab,ti,kw) AND ('Cone Beam Computed Tomography'/exp OR 'cone-beam':ab,ti,kw  OR 'x-ray computed tomography'/exp OR 'x-ray computed tomography':ab,ti,kw OR 'CBCT':ab,ti,kw) NOT  ('case report*':ti ) AND ('german':la OR 'english':la) NOT (animals/exp NOT humans/exp) | 105 |

Cochrane Central Register of Cochrane Reviews and Controlled Trials (CENTRAL) (1992 to September 01, 2025)

| Query | Results |
| --- | --- |
| ([mh "Sleep Apnea, Obstructive"] OR (Obstructive sleep apnea*):ti,ab,kw OR (Obstructive sleep apnoea*):ti,ab,kw OR (OSA):ti,ab,kw OR (OSAHS):ti,ab,kw OR (SRBD):ti,ab,kw OR (sleep related breathing disorder):ti,ab,kw OR (sleep-disordered breathing):ti,ab,kw OR (SDB):ti,ab,kw OR (Sleep Apnea Hypopnea*):ti,ab,kw OR (Sleep Apnoea Hypopnoea*):ti,ab,kw OR (Upper Airway Resistance Sleep Apnea*):ti,ab,kw OR (Upper Airway Resistance Sleep Apnoea*):ti,ab,kw) AND ([mh "Dentistry"] OR [mh "Dentists"] OR (dentist*):ti,ab,kw OR (dental*):ti,ab,kw) AND ([mh "Cone-Beam Computed Tomography"] OR (cone-beam):ti,ab,kw OR [mh "tomography, x ray computed"] OR (tomography, x ray computed):ti,ab,kw OR (CBCT):ti,ab,kw) NOT ((case report*):ti ) NOT ([mh animals] NOT [mh humans]) | 20 |

Web of Science Core Collection (WOS.SCI: 1900 to 2025; WOS.AHCI: 1975 to 2025; WOS.ESCI: 2020 to 2025; WOS.SSCI: 1956 to 2025) (to September 01, 2025)

| Query | Results |
| --- | --- |
| (TS=Obstructive sleep apnea* OR TS=Obstructive sleep apnoea* OR TS=OSA OR TS=OSAHS OR TS=SRBD OR TS=sleep related breathing disorder OR TS=sleep-disordered breathing OR TS=SDB OR TS=Sleep Apnea Hypopnea* OR TS=Sleep Apnoea Hypopnoea* OR TS=Upper Airway Resistance Sleep Apnea* OR TS=Upper Airway Resistance Sleep Apnoea*) AND (TS=dentist* OR TS=dental*) AND (TS=cone-beam OR TS=X-Ray Computed Tomography OR TS=Computed X Ray Tomography OR TS=CBCT) NOT (TI=case report*) AND (LA=German OR LA=English) | 57 |

ClinicalTrials.gov (to September 01, 2025)

| Query | Results |
| --- | --- |
| (Obstructive Sleep Apnea OR OSA OR OSAHS OR SRBD OR sleep related breathing disorder OR sleep-disordered breathing OR SDB OR Sleep Apnea Hypopnea OR Sleep Apnoea Hypopnoea OR Upper Airway Resistance Sleep Apnea OR Upper Airway Resistance Sleep Apnoea) AND (Dentistry OR Dentists OR dentist OR dental) AND (cone-beam OR CBCT ) NOT (case report OR case reports) | 9 |

**Appendix II** Reported sleep parameters used across included studies - abbreviation, alternative designation and definition

| **Abbreviation** | **Alternative designation** | **Definition** |
| --- | --- | --- |
| AHI | Apnea-Hypopnea-Index | Apneas and hypopneas per hour of sleep |
| Supine AHI | Positional AHI | Number of apneas and hypopneas per hour of sleep in supine position |
| AI | Apnea Index | Number of apneas per hour of sleep |
| CAHI | Central Apnea-Hypopnea-Index | Number of central apneas and hypopneas per hour of sleep |
| CAI | Central Apnea Index | Number of central apneas per hour of sleep |
| OAHI | Obstructive Apnea-Hypopnea-Index | Number of obstructive apneas and hypopneas per hour of sleep |
| ODI | Oxygen Desaturation Index, Sleep Desaturation Index (SDI) | Number of oxygen desaturations (≥ 3%) per hour of sleep |
| RDI | Respiratory Disturbance Index, Respiratory Disorder Index | Number of apneas, hypopneas and respiratory effort-related arousals (RERAs) per hour of sleep |
| REM | Rapid eye movement sleep percentage | Percentage of total sleep time spent in REM sleep |
| Snore index |  | Number of snoring events per hour of sleep |
| Snoring (%) |  | Proportion of total sleep time spent snoring |
| SpO₂ | Oxygen saturation | Percentage of peripheral arterial oxygen saturation |
| SpO₂ < 90% | Sleep time in % below 90% SpO₂ | Percentage of sleep time spent below 90% oxygen saturation below 90% |
| Min. SpO₂ | Minimal SpO₂, minimum average overnight arterial oxygen saturation, minimum oxygen saturation | Minimal oxygen saturation during sleep |
| Mean SpO_2_ | Mean saturation | Mean arterial oxygen saturation during sleep |
| Total SpO_2_ | Total number of desaturations | Absolute number of oxygen desaturation events during sleep |
| SWS | Slow wave sleep percentage | Percentage of total sleep time spent in N3 sleep stage |

**Appendix III** Reported CBCT measuring variables of airway dimension used across included studies - abbreviation, alternative designation and definition

| **Abbreviation** | **Alternative designation** | **Definition** |
| --- | --- | --- |
| RP region | Retropalatal region | Airway segment extending from the hard palate /posterior nasal spine (PNS) to the tip of the uvula |
| minAP (RP) | Minimum anteroposterior diameter, anteroposterior dimension (RP) | Smallest anteriorposterior dimension of RP region |
| L, minLAT | Minimum lateral diameter (RP) | Smallest lateral dimension of RP region |
| RPO | Minimum retropalatal area | Smallest cross-sectional area within the retropalatal region |
| - | Retroglossal region | Airway segment extending from the tip of the uvula to the base of the epiglottis |
| RGO | Minimum retroglossal area | Smallest cross-sectional area within the retroglossal region |
| TAV | Total airway volume, volume, volume of the upper airway | Total volume of oropharyngeal airway (RP+RG = From PNS to tip of epiglottis) |
| CSAmin | minimum cross-sectional area, minimum airway area, slice with smallest computed airway-area | Smallest cross-sectional area of the upper airway lumen |
| AP diameter at CSAmin | Sagittal diameter at minimum cross-section of airway lumen | Sagittal diameter at the minimal cross-sectional area |
| L diameter at CSAmin | Lateral diameter at the minimal cross-sectional area | Transverse diameter at the minimal cross-sectional area |
| TAL | Total airway length, airway length, length of the upper airway | Distance from a plane through the PNS (parallel to FH) to a plane through the base of the epiglottis (parallel to FH) |
| CSAavg | Average cross-sectional area | Average cross-sectional area of the upper airway |
| CSAmin/CSAavg ratio | Airway uniformity index, uniformity of volume, shape of upper airway | Ratio of minimum to average cross-sectional area |
| VP | Velopharynx | Upper airway segment from PNS to uvula tip |
| OP | Oropharynx | Upper airway segment from uvula tip to epiglottis base |
| OPV | Oropharynx volume | Volume defined by points: basion, PNS, hyoid, and inferior-anterior edge of C2 vertebra |
| NP | Nasopharynx volume | Volume/area of nasopharynx: borders from sella to PNS and basion to PNS |
| _ | Nasopharynx cross-sectional area | Cross-sectional area at the roof of nasopharynx, typically at hard palate level (8 mm slice) |
| _ | Retropalatal high cross-sectional area | Cross-sectional area in an 8 mm slice distal to nasopharyngeal CSA |
| _ | Retropalatal low cross-sectional area | Cross-sectional area in an 8 mm slice distal to the retropalatal high CSA |
| _ | Retroglossal cross-sectional area | Cross-sectional area in an 8 mm slice distal to the retropalatal region, limited by epiglottis tip 8mm thick anatomical slice |
| VA | Velopharyngeal area | Cross-sectional area of velopharynx at uvula tip |
| HA | Hypopharyngeal area | Cross-sectional area at floor of vallecula |

**Appendix IV** Reported CBCT variables of skeletal measurements - abbreviation, alternative designation and definition

| **Abbreviation** | **Alternative designation** | **Definition** |
| --- | --- | --- |
| _ | Maxillomandibular enclosure size | Area enclosed by hyoid, mandible, incisors, maxilla, and anterior boundary of C2–C3 |
| AMT | Anterior mandibular thickness |  |
| I-SGT | Lower incisor - genial tubercle distance | Distance from lower incisor tip to superior genial tubercle |
| IGT-IBM | Inferior genial tubercle -mandibular base distance | Distance from inferior genial tubercle to mandibular base |
| GH | Genial tubercle height | Vertical height of genial tubercle |
| GW | Genial tubercle width | Horizontal width of genial tubercle |
| LFD | Lingual foramen diameter | Diameter of lingual foramen |
| LCL | Lingual canal length | Length of lingual canal |
| LVDL | Lingual foramen - mandibular base distance | Vertical distance from lingual foramen to mandibular base |
| LVDA | Lingual foramen - alveolar crest distance | Vertical distance from lingual foramen to alveolar crest |
| MP-H, H-MP | Hyoid–mandibular plane distance | Perpendicular distance from most antero-cranial hyoid point to mandibular plane |
| SNA | Maxilla - cranial base angle | Sagittal relation of the maxilla to the anterior cranial base |
| SNB | Mandible - cranial base angle | Sagittal relation of the mandible to the anterior cranial base |
| ANB | Maxillo - mandibular angle | Sagittal relation between mandible and maxilla |
| SN-PP | Palatal plane angle | SN plane to palatal plane measured on mid-sagittal slice |
| SN-DSP | Distal segment plane angle | SN plane to plane passing through bilateral lingual foramen and bilateral mental foramen on mid-sagittal slice |
| SN-MP | Mandibular plane angle | SN plane to Go-Me plane |
| MMV | Maxillomandibular volume | Polyhedron connecting bilateral condylion, bilateral gonion, ANS and menton |
| ANS-PNS | Maxillary length | Linear distance from anterior nasal spine (ANS) to posterior nasal spine (PNS) |
| Me-Go | Mandibular length | Mandibular body length from Gonion to Menton |
| MdL | Mandibular length | Distance from condylion to B on mid-sagittal slice |
| MxL | Maxillary length | Distance condylion to A |
| MxW | Maxillary width | Distance from left & right greater palatine foramen to mid-sagittal plane |
| MxAW | Maxillary alveolar width | Distance from left & right alveolar bone from 1st molar bifurcation to mid-sagittal plane |
| MxBW | Maxillary basal width | Distance from left and right most outer cortical bone extending at palatal level to mid-sagittal plane |
| MxICW | Maxillary intercanine width | Distance between upper canine cusps and midsagittal plane |
| MxIMW | Maxillary intermolar width | Distance between upper first molar mesiobuccal cusps and midsagittal plane |
| MdW | Mandibular width | Distance between right and left mental foramina to midsagittal plane |
| IGoW | Intergonial width | Distance between right and left gonion |
| MdICW | Mandibular intercanine width | Distance between lower canine cusps and midsagittal plane |
| MdIMW | Mandibular intermolar width | Distance between lower first molar mesiobuccal cusps and midsagittal plane |
| H-S | Hyoid - sella distance | Distance from hyoid to sella |
| H-GT | Hyoid - genial tubercle distance | Distance from hyoid to genial tubercle |
| H-C3 | Hyoid - C3 distance | Distance from hyoid to most anterior-inferior point of C3 |

**Appendix V** Reported CBCT variables of soft tissue measurements across included studies - abbreviation, alternative designation and definition

| **Abbreviation** | **Alternative designation** | **Definition** |
| --- | --- | --- |
| TGL | Tongue length | Distance from tongue tip to epiglottis base |
| TGH | Tongue height | Maximum vertical dimension perpendicular to tongue length |
| ANSV | Anterior neck space volume | Volume of polyhedron bounded by bilateral soft tissue gonion, menton, and cervicomental junction |
| PNS-UT, PMU, P-pm, SPL | Soft palate length | Linear distance from PNS to the uvula tip (UT) |
| HSP | Horizontal soft palate length | Distance from PNS to vertical line through posterior soft palate |
| VSP | Vertical soft palate length | Distance from horizontal line through PNS to tip of soft palate |
| Max-Sp, x-y, MPT | Soft palate thickness | Maximum thickness of soft palate perpendicular to PNS–UT line |
